# Nirsevimab for preventing respiratory syncytial virus lower respiratory tract infections in infants: a systematic review and meta-analysis

**DOI:** 10.3389/fpubh.2025.1641085

**Published:** 2025-10-24

**Authors:** Samira Soudani, Lorenzo Bertizzolo, Mehdi Ghemmouri, Mary Chappell, Rachael McCool, Katie Reddish, Paul Miller, Erin Barker, Harriet Fewster

**Affiliations:** ^1^Sanofi, Global Health Economics and Value Assessment, Lyon, France; ^2^Sanofi, Global Medical, Lyon, France; ^3^York Health Economics Consortium, University of York, York, United Kingdom

**Keywords:** nirsevimab, observational studies, systematic review, meta-analysis, RSV

## Abstract

**Background:**

Respiratory syncytial virus (RSV) causes lower respiratory tract infections (LRTIs) in infants, leading to substantial morbidity and mortality. Nirsevimab, a long-lasting monoclonal antibody, has been demonstrated to reduce RSV-related outcomes in randomized controlled trials (RCTs) and real-world settings. The object was to review the existing real-world evidence (RWE) on the effectiveness of nirsevimab in preventing RSV-LRTIs.

**Methods:**

Searches of six databases in addition to trial registries, HTA/regulatory agency webpages and conference abstracts were conducted in November 2024. Observational studies evaluating nirsevimab prophylaxis in infants during their first RSV season or high-risk infants in their second season were included. For outcomes evaluated by more than one study, feasibility assessment was conducted and, where appropriate, studies were combined in meta-analyses.

**Results:**

Sixteen studies reporting effectiveness outcomes were included. The studies were conducted across four countries (Spain, Italy, US and France), and included 141,550 infants. Nirsevimab showed significant effectiveness for preventing RSV-LRTI hospitalization (reduction in risk from hazard ratios and odds ratios of 84.5%; 95% CI: 73.6%−90.9%; *I*^2^: 0 and 73.7%; 95% CI: 42.3%−88.0%; *I*^2^: 0%, respectively), intensive care unit (ICU) admission (85.9%; 95% CI: 13.2%−97.7%) and ventilatory support (87.1%; 95% CI: 70.2%−94.4%). Nirsevimab was also effective in preventing RSV-LRTI visits in primary care (75.8%; 95% CI: 40.4%−92.7%) and emergency departments (87.9%; 70.3%−95.1%).

**Conclusions:**

Real-world evidence confirms the effectiveness of nirsevimab against RSV-LRTIs and underlines the public health impact of the intervention in preventing RSV-related health outcomes. There is no clear evidence that effectiveness differs for infants born in season (at birth immunization) compared with out of season groups, or that effectiveness varies in preterm infants compared to healthy term infants.

**Systematic review registration:**

https://www.crd.york.ac.uk/PROSPERO/view/CRD42024591323.

## 1 Introduction

Respiratory syncytial virus (RSV) is a highly prevalent virus that typically causes cold-like symptoms (1) and can, in some cases, cause lower respiratory tract infections (LRTIs). RSV LRTIs are a leading cause of severe respiratory illness, particularly in infants and young children (1). These infections are associated with substantial morbidity and can result in hospitalizations, especially in high-risk groups such as premature infants, those with underlying medical conditions (e.g., congenital heart disease or chronic lung disease), and immunocompromised individuals (2).

Preventative treatments for RSV LRTIs include monoclonal antibodies like nirsevimab and palivizumab, designed to provide protection by targeting the F protein of the virus (3). Palivizumab is licensed for use in high-risk infants and requires monthly injections due to its short half- life (4), whilst nirsevimab provides longer-lasting protection. Nirsevimab is licensed for the first RSV season for all infants (<12 months), and the second season for the most high-risk groups (3). It is approved in over 50 countries, including the UK, EU and USA (3, 5). In the USA, nirsevimab is recommended for all infants under 8 months of age and, for children at high risk for severe disease entering their second RSV season, up to 19 months (6). In the EU, it is recommended for all infants in their first season, and for children up to 24 months of age who remain vulnerable to severe RSV disease in their second RSV season (7). In the UK, it is recommended for high-risk infants and children, up to 24 months of age (8).

Nirsevimab provides passive immunity by directly supplying pre-formed antibodies. It works by binding to a highly conserved site on the RSV prefusion F protein, an essential component for the virus to enter and infect cells (9). This binding action neutralizes the virus and prevents cell-to-cell fusion, effectively blocking the infection at the cellular level. It can be administered before the RSV season, or at birth during the season, as a single 50 or 100 mg dose (depending on body weight) (10).

A maternal vaccine is also available for RSV prevention, providing newborns protection from their immunized mothers (11). This bivalent RSV prefusion F protein-based (RSVpreF) vaccine, has shown 68% effectiveness against RSV-associated acute respiratory illness (12). However, maternal vaccination effectiveness can fluctuate if the vaccination occurs too late in the pregnancy for adequate transplacental antibody transfer or too early before the RSV season. This is particularly relevant for infants born prematurely or born before the RSV season for year round RSVpreF vaccination (13).

Several RCTs have been conducted to evaluate nirsevimab for the prevention of RSV LRTIs in infants (5, 10, 14, 15). These studies have shown that nirsevimab significantly reduces RSV-related medical visits and hospitalizations in a range of pediatric populations, including preterm (born <35 weeks gestational age) and full-term infants (5, 10, 14), and infants with specific high-risk conditions (15). The trials demonstrated consistent protection against RSV across various geographies and healthcare settings, with protection lasting at least 6 months (16).

While clinical trials provide controlled evidence of efficacy, observational real-world studies are essential to understand how nirsevimab performs in everyday clinical practice. Therefore, the objective of this systematic review is to consolidate and summarize the evidence from real-world studies, focusing on the effectiveness of nirsevimab in preventing RSV LRTIs.

## 2 Methods

### 2.1 Eligibility criteria

Predefined eligibility criteria were documented in a protocol (see Supplementary material 1). Studies eligible for this systematic review were observational studies (comprising cohort, case-control and cross-sectional studies) of nirsevimab prophylaxis in infants (≤1 year) in their first RSV season, or children in their second RSV season with high-risk conditions (such as congenital heart diseases with significant hemodynamic involvement, bronchopulmonary dysplasia, severe immunosuppression, inborn errors of metabolism, neuromuscular disorders, severe lung diseases, genetic syndromes with relevant respiratory problems, Down syndrome, cystic fibrosis, and people in palliative care).

Studies reporting outcomes related to RSV-LRTIs were eligible. These included specific RSV-LRTIs outcomes as well as outcomes associated with RSV-LRTIs, for example, all-cause LRTIs, RSV-acute respiratory infection (ARI) and RSV-bronchiolitis outcomes. Studies using different definitions of outcomes were included, for example, where disease was defined using ICD codes or where it was diagnosed clinically. Nirsevimab coverage was also included as an outcome. No date or language limits were used, except that conference abstracts published prior to 2021 were excluded.

### 2.2 Searches

Searches for eligible studies were conducted between 16 and 17 July 2024 and updated on 26 November 2024 in the following databases: Medline, Embase, Cochrane Database of Systematic Reviews (CDSR), Cochrane Central Register of Controlled Trials (CENTRAL), HTA Database and Conference Proceedings Citation Index—Science (CPCI-S). Searches of trial registries and HTA/regulatory agency webpages were also conducted, as well as non-database conference searches for key conferences. Full search strategies and details of the resources searched are shown in Supplementary material 2. In addition to literature searches, the included studies list of any retrieved relevant systematic reviews published in the last 3 years were checked for additional eligible studies.

### 2.3 Study selection, data extraction and risk of bias assessment

Two reviewers (MC and KR) independently assessed the title/abstracts and full texts of retrieved records, with any disagreements resolved by discussion. For studies reporting effectiveness data, study methods, patient characteristics and outcome data were extracted into a Microsoft Excel template. Data extraction was conducted by one reviewer, with every data point checked by a second reviewer. Risk of bias assessment was undertaken with the Joanna Briggs Institute (JBI) checklists for cohort and case control studies (17). An overall assessment of risk of bias was made. This was not based on a count of affected domains, but determined with reference to the overall severity of identified biases. One reviewer conducted risk of bias assessment, and all judgements were checked by a second reviewer.

### 2.4 Feasibility assessment

Studies that reported outcomes in terms of “effectiveness” (1-effect measure ×100%), or provided data allowing calculation of “effectiveness,” were considered for inclusion in the meta-analysis. These studies went forward to a feasibility assessment.

The feasibility of combining studies in meta-analysis was assessed by comparing study and patient characteristics across studies reporting the same outcomes. Studies were first mapped according to outcome to identify any potential meta-analyses. For these outcomes, the feasibility assessment compared the similarity of the following key characteristics that were identified as potential treatment effect modifiers: study design, methods for case identification and definition, comparator, duration of follow-up/length of study, inclusion and exclusion criteria, age, proportion preterm and timing of nirsevimab administration.

### 2.5 Analysis

For outcomes with more than one contributing study, following feasibility assessment, generic inverse variance meta-analyses were performed using fixed and random effects models. Analysis was performed in R using the “meta” package (18). Eight outcomes were included in the feasibility assessment and all proceeded to meta-analysis {RSV-ARI hospitalization, RSV-LRTI hospitalization [odds ratio (OR)-derived effectiveness], RSV-LRTI hospitalization [hazard ratio (HR)-derived meta-analysis], RSV-LRTI intensive care unit (ICU) admission, RSV-bronchiolitis hospitalization, RSV-bronchiolitis intensive care unit (ICU) admission, RSV-bronchiolitis requiring ventilation and 0–3 vs. 3–6 months subgroups for RSV-bronchiolitis hospitalization}.

For meta-analyses with two contributing studies where there was no evidence of statistical heterogeneity, the fixed effect model was treated as the main analysis and the random effects model as a sensitivity analysis (19). Otherwise, the random effects model was treated as the main analysis and the fixed effect model as a sensitivity analysis. In the random effects meta-analyses, Hartung-Knapp and Sidik-Jonkman adjustments were made. Heterogeneity was assessed using the chi-squared and *I*-squared statistics. Due to the small sample sizes, robust detection of outliers or publication bias could not be performed. We conducted sub-group meta-analysis where data for pre-specified variables was available: pre-term vs. healthy term infants and infants receiving nirsevimab at birth vs. at catch up. Related to this, we also conducted a subgroup analysis comparing infants receiving nirsevimab at 0–3 vs. 3–6 months old.

## 3 Results

### 3.1 Results of the searches

**Figure 1:**
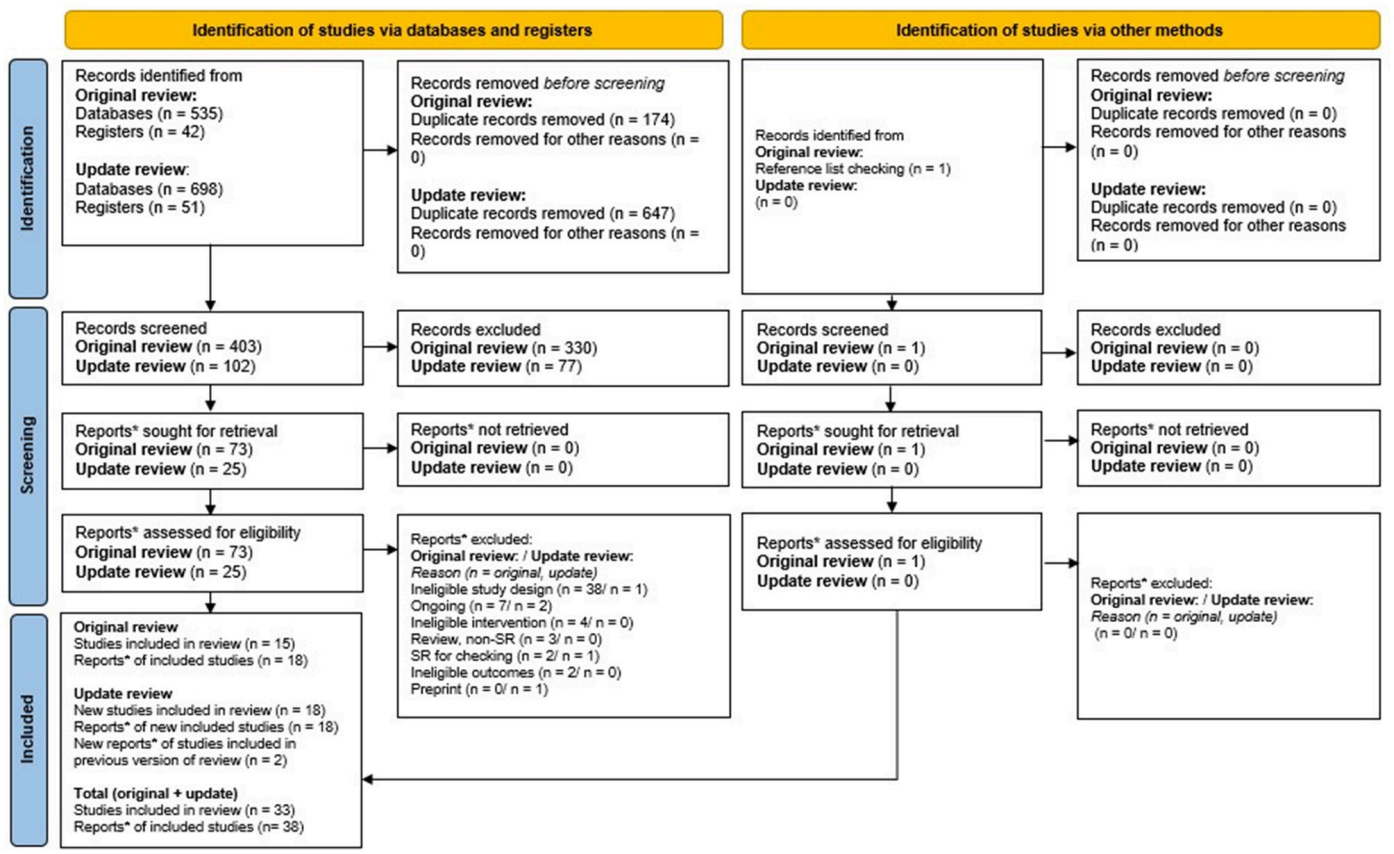
PRISMA.

Five hundred five unique records were identified from the searches, and 1 record was found through reference checking of previous relevant systematic reviews (Figure 1). 407 of these were excluded at title/abstract and 60 were excluded at full-text screening. 33 studies, reported in 38 publications met review eligibility criteria. Of these 16 (seven cohort studies and nine case control studies) provided effectiveness data.

### 3.2 Included study characteristics

The study methods and characteristics of patients in the included studies are shown in Tables 1, 2, respectively. All studies were conducted in the 2023/2024 RSV season.

**Table 1:** Study methods.

**Study**	**Trial number**	**Details of funding bodies**	**Location [sites (*n*)/countries]**	**Case definition**	**Comparator**	**Date of trial data**	**Duration of follow-up**
**Cohort studies**							
Ares-Gómez, 2024 (20 )	NCT06180993	Sanofi and AstraZeneca	14 public hospitals, Galicia, Spain	Admitted with LRTI with positive RSV laboratory test results (records reviewed by public health specialists)	Admitted with RSV-LRTI but not receiving nirsevimab	September 2023 or birth to December 2023	Up to 3 months. Median 81 days (IQR: 68–87)
Coma, 2024 (21)	NR	None	Five Catalan health databases, Catalonia, Spain	Admitted with RSV-bronchiolitis (measured as positive rapid antigen test) in primary care or ICU	Admitted with RSV-bronchiolitis but not receiving nirsevimab	October 1 2023–January 31 2024	Up to 4 months
Consolati, 2024 (22)	NR	Azienda Usl Valle d'Aosta, Italy	Number of sites NR, Valle D'Aosta region, Italy	Admitted for RSV bronchiolitis (data from Local Health Unit information systems). RSV confirmation unclear	Admitted with RSV-bronchiolitis but not receiving nirsevimab	December 20 2023–February 15 2024	Up to 8 weeks
Barbas Del Buey, 2024 (23)	NR	None	Number of sites NR, Madrid, Spain	Prospective collection of primary care and hospital data by epidemiological surveillance. RSV-ARI confirmed by PCR or the antigen test or RSV isolation test of a respiratory sample	Eligible infants not receiving nirsevimab	1 October 2023–29 February 2024	Up to 5 months
Estrella-Porter, 2024 (24)	NR	NR	Number of sites NR, Valencia, Spain	Positive RSV cases (data from the Microbiological Surveillance Network of the Valencian Community) or admission for RSV-ARIs (data from Alumbra database)	Infants RSV positive or admitted for RSV-ARIs not receiving nirsevimab	October 2023–January 9 2024	Up to 14 weeks
Ezpeleta, 2024 (25)	NR	Instituto de Salud Carlos III with the European regional development fund	Number of sites NR, Navarre, Spain	Admitted to ICU or attending ER for LRTI testing positive for RSV with PCR assay (data from epidemiological surveillance systema)	Admitted to ICU or attending ER for RSV-LRTI, not receiving nirsevimab	October 2023–28 January 2024	Up to 4 months
Hsiao, 2024 (26)	NR	Sanofi and AstraZeneca	NR but authors are from France and the US (California and Pennsylvania)	No details of source of data. Data was for medical encounters for RSV-LRTIs in any setting	Eligible infants not receiving nirsevimab	NR “between 2023 and 2024”	NR
**Case-control studies**							
Aguera, 2024 (28 )	NR	NR	Three hospitals, Barcelona, Catalonia and Andorra	Admitted for ≥ 24 h for LRTI testing positive for RSV using polymerase chain reaction (PCR)-based tests	Admitted for at least 24 h for LRTI but testing negative for RSV	November 2023–February 2024	NA
Assad, 2024 (29)	NCT06030505	National agency for AIDS research and ATIP-Avenir PROGRAM	Six hospitals, France	Admitted with bronchiolitis testing positive for RSV with PCR assay	Infants with clinical visits to the same hospitals for conditions unrelated to RSV infection	October 15–December 10 2023	NA
Carbajal, 2024 (30)	NCT06185647	None	One ED in Paris, France	Infants with a diagnostic code for bronchiolitis or presenting with respiratory signs from medical records. RSV confirmed by RT-PCR in some hospitalized infants. For others, RSV status imputed using a Bayesian logistic model to predict RSV positivity	Infants visiting pediatric ED for bronchiolitis, without bronchiolitis or upper respiratory tract infections	October 14, 2023–29 February 2024	NA
Lassoued, 2024 (31)	NCT04471493	Association Clinique et Thérapeutique Infantile du Val de Marne, French Pediatrician Ambulatory Association, GSK, MSD, Pfizer and Sanofi	Surveillance system with 107 pediatricians/France	Outpatients aged < 12 months who had a diagnosis of bronchiolitis who were RSV positive on a rapid antigen test	Infants eligible for nirsevimab visiting ambulatory pediatrician for bronchiolitis, testing negative for RSV	September 15 2023–February 1 2024	NA
Lefferts, 2024 (32)	NR	NR	One hospital, Alaska, USA	Infants with an outpatient visit or hospitalization for ARI with discharge diagnosis code and RSV RNA testing	Infants eligible for nirsevimab with medically attended ARI, testing negative for RSV	October 1 2023–end of June 2024	NA
Lopez-Lacort, 2024 (33)	NR	Instituto de Salud Carlos III and the European Union	Five hospitals in Valencia, three hospitals in Murcia, one in Valladolid	Admitted with LRTI testing positive for RSV with multiplex RT-PCR (unclear whether this applied to all included infants)	In the case control study, patients Admitted for LRTI testing RSV-negative	October 1 2023–between December 31 2023 and January 10 2024, depending on the hospital	NA
Lopez-Lacort, 2024 (34)	NR	Instituto de Salud Carlos III and the European Union	57 primary care centers/Valencia and Murcia, Spain	Prospectively enrolled infants with ≥ sign of lower respiratory tract disease or apnoea, testing positive for RSV on PCR	Infants eligible for nirsevimab with medically attended LRTI, testing negative for RSV	November 1st, 2023 and February 29th, 2024	NA
Moline, 2024 (35)	NR	NR	Seven pediatric academic medical centers, US	Admission for ARI, testing positive with PCR	Admitted for ARI, testing negative for RSV	October 1 2023–February 29 2024	NA
Paireau, 2024 (36)	NR	Santé publique France and the Laboratoire d'Excellence Integrative Biology of Emerging Infectious Diseases program	20 pediatric intensive care units (PICUs), France	Admission to pediatric ICU for bronchiolitis, testing positive for RSV on PCR	Admission to pediatric ICU for bronchiolitis, testing negative for RSV	September 15 2023 to January 31 2024	NA

AIDS, acquired immunodeficiency syndrome; ARI, acute respiratory infection; ED, emergency department; ER, emergency room; ICU, intensive care unit; IQR, interquartile range; LRTI, lower respiratory tract infection; NA, not applicable; NR, not reported; PCR, polymerase chain reaction; RNA, ribonucleic acid; RSV, respiratory syncytial virus; RT-PCR, reverse transcription polymerase chain reaction.

**Table 2:** Population characteristics.

**Study**	**Group**	***N***	**Key inclusion criteria**	**Key exclusion criteria**	**Gender *n* (%) [male]**	**Age [months]**	**Preterm *N* (%)**	**Timing**	**Coverage (%)**
**Cohort studies**									
Ares-Gómez, 2024 (20 )	Nirsevimab	9,408	Infants born from April 1 to Dec 15, 2023, eligible for nirsevimab	NR	4,751 (51)	MeanV (SD) 4.14 (2.44)	616 (6.5)	Birth: 3,340 (33%) Catch-up: 6,919 (67%)	At birth: 95.4 Catch-up: 89.9
	No Nirsevimab	851			448 (53)	Mean (SD) 5.05 (2.29)	40 (4.7)	NA	
Coma, 2024 (21)	Nirsevimab	23,127	Infants born between April and September 2023 in Catalonia eligible for nirsevimab	No valid health ID no., died/moved away, not assigned to participating practice	11,916 (52)	Median (IQR) days 88 (44–134)	NR	Given at birth or catch up (proportions NR)	87.2
	No nirsevimab	3,398			1,739 (51)	Median (IQR) days 106 (52–151)	NR	NA	
Consolati, 2024 (22)	Nirsevimab	369	Infants born between 1 May 2023 and 15 February 2024 and eligible for nirsevimab	Pre-existing risk factors and had already received palivizumab	NR	NR	NR	Birth: 77 (21%), Catch-up: 292 (79%)	68.7
	No Nirsevimab	168			NR	NR	NR	NA	
Barbas Del Buey, 2024 (23)	Nirsevimab	29,684	Infants born from April 1 2023 to December 31, 2023	Death, change in residence outside area and losses before follow-up, stillbirths or abortions, non-resident parents, transient population, duplicates/recording errors. Palivizumab or vaccination of mother	15,386 (51.83)	Median (IQR) 0.98 (3.38)	2,220 (7.48)	At birth or catch up: proportions NR	80.1
	No nirsevimab	7,383			3,731 (50.54)	Median (IQR) 2.85 (3.51)	463 (6.27)	NA	
Estrella-Porter, 2024 (24)	Nirsevimab	24,223	NR	NR	12,351 (51)	NR	4,039 (16.7)	Birth: 9,124 (38%), Catch up: 15,099 (62%)	At birth: 92.1 Catch-up: 86.5
	No nirsevimab	3,139			1,638 (52)	NR	528 (16.8)	NA	
Ezpeleta, 2024 (25)	Nirsevimab	1,083	NR	NR	583 (54)	NR	NR	Birth: 1,035 (96%), immunization delayed: 48 (4%)	92.0
	No nirsevimab	94			49 (52)	NR	NR	NA	
Hsiao, 2024 (26)	Nirsevimab	15,647	Healthy-term infants born in April 2023 or later	Infants with high-risk conditions or whose mothers were RSV-vaccinated	NR	NR	NR	At birth or catch up: proportions NR	NR
	No nirsevimab	16,253			NR	NR	NR	NA	
**Case-control studies**									
Aguera, 2024 (28 )	Total population	234	Infants ≤ 12 months, admitted for bronchiolitis for ≥24 h, tested for RSV with PCR	Infants who only underwent an antigen-detection-based test, or had a previous episode of bronchiolitis or LRTI	139 (59)	Median (IQR) 3.6 (1.5–8.1)	39 (17)	Given at birth or catch up (proportions NR)	NA
Assad, 2024 (29)	Cases	690	Infants ≤ 12 months admitted for RSV-bronchiolitis (by PCR)	Infants who had previously received palivizumab and those whose mother had been vaccinated against RSV during pregnancy	357/687 (52.0)	Median (IQR) 3.1 (1.8–5.3)	38/665 (5.7)	Given at birth or catch up (proportions NR)	NA
	Controls	345	Infants ≤ 12 months visiting PED for conditions unrelated to RSV infection. Matched to cases (2:1) according to age, date of hospital visit, and participating center	NR	196/343 (57.1)	Median (IQR) 3.4 (1.6–5.6)	21/306 (6.9)		
Carbajal, 2024 (30)	Cases	864	Infants aged ≤ 12 months attending pediatric emergency department for whom nirsevimab status was known	Infants with upper respiratory tract infections were not included in the main analysis included in a sensitivity analysis	≤3 months: 130 (60); 3–6 months: 180 (57); 6–12 months: 200 (60)	≤3 months: 217 (14.7); 3–6 months: 315 (36.5); 6–12 months: 332 (38.4)	NR	At birth or catch up: proportions NR	NA
	Controls	1922			≤ 3M: 389 (54); 3–6 M: 187 (57); 6–12 M: 483 (55)	≤ 3M: 723 (37.6); 3–6 M: 327 (17.0); 6–12 M: 872 (45.4)	NR		
Lassoued, 2024 (31)	Cases	453	Infants < 12 months with diagnosis of bronchiolitis visiting ambulatory pediatrician for whom an RSV rapid antigen test was performed	Infants in the HARMONIE study, previous immunization with palivizumab or maternal vaccination against RSV	269 (59.4)	< 3 months: 53 (11.7); 3–6 months: 126 (27.8); >6 months: 274 (60.5)	32/358 (8.9; before 37 weeks)	At birth or catch up: proportions NR	NA
	Controls	430			279 (64.9)	< 3M; 35 (8.2); 3–6 M: 164 (38.1); >6M: 231 (53.7)	33/352 (9.4; before 37W)		
Lefferts, 2024 (32)	Cases	68 [39 (57%) in 1st RSV season]	Infants < 20 months, with outpatient visit or hospitalization for acute respiratory illness (ARI)	Received nirsevimab < 7 days earlier, or received >1 dose of nirsevimab on different dates, 1 dose of palivizumab, mother had received RSV vaccine during pregnancy, negative RSV test result but had RSV discharge code, ineligible for nirsevimab	38 (56)	0–5 months: 22 (32) 6–11 months: 16 (24; data for children in 1st and 2nd RSV season)	NR	At birth or catch up: proportions NR	NA
	Controls	404 [253 (63%) in 1st RSV season]			214 (53)	0–5 months: 135 (33) 6–11 months: 117 (29; data for children in 1st and 2nd RSV season)	NR		
Lopez-Lacort, 2024 (33)	Total population	166	Infants eligible for nirsevimab born from 1 April 2023	NR	NR	126 (76%) 0–3 months	NR	Given at birth or catch up (proportions NR)	Valencia: 89.8 Murcia: 88.9 Valladolid: 98.6
Lopez-Lacort, 2024 (34)	Cases	44	Infants born after April 1st, 2023, attending primary care centers with symptoms of LRTI, detection of RSV through RT-PCR and the presence of at least one sign of lower respiratory tract disease or apnoea	Symptom onset exceeded 10 days	30 (68)	Mean SD: 4.55 (2.04) 0–3 months: 15 (34%) 3–6 months: 29 (66%)	3 (6.8)	Eligible at birth: 7 (16%); Catch-up: 37 (84%). Received at 0 months: 15 (45%)	NA
	Controls	116			72 (62)	Mean SD: 4.62 (2.33); 0–3 M: 38 (33%); 3–6 M: 78 (67%)	15 (13)	Eligible at birth: 25 (22%); Catch-up: 91 (78%). Received at 0M: 32 (30%)	
Moline, 2024 (35)	Cases	407	Infants aged < 8 months as of October 1, 2023, or born after October 1, 2023; verified nirsevimab status, reported gestational age at birth, medical record review to assess for underlying conditions, testing positive for RSV	Enrolled before nirsevimab became available, received palivizumab, reported maternal RSV vaccination during pregnancy, inconclusive/unknown RSV test results	225 (55)	< 1 month: 51 (13%) 1–4 months: 234 (57%) 5–8 months: 116 (28%) 9–10 months: 6 (1%)	77 (19)	Given at birth or catch up (proportions NR)	NA
	Controls	292	As above, testing negative for RSV		181 (62)	< 1 month: 60 (21%) 1–4 months: 111 (38%) 5–8 months: 101 (34%) 9–12 months: 20 (7%)	69 (24)		
Paireau, 2024 (36)	Cases	238	Infants < 2 years requiring PICU admission for severe bronchiolitis, testing positive for RSV	Administration of palivizumab, un-known comorbidities/ prematurity/sex, received nirsevimab < 8 days prior to hospitalization, date of nirsevimab administration unknown	123 (52)	0–3 months: 225 (95%) 4–8 months: 13 (5%)	23 (10)	Given at birth or catch up (proportions NR)	NA
	Controls	50	As above, testing negative for RSV		34 (68)	0–3 months: 38 (76%) 4–8 months: 12 (24%)	16 (32)		

ARI, acute respiratory infection; IQR, interquartile range; LRTI, lower respiratory tract infection; NA, not applicable; NR, not reported; PC, pediatric care; PCR, polymerase chain reaction; PED, pediatric emergency department; PICU, pediatric intensive care unit; RSV, respiratory syncytial virus; RT-PCR, reverse transcription polymerase chain reaction; SARI, severe acute respiratory infection; SD, standard deviation; YKHC, Yukon-Kuskokwim Health Corporation.

Seven cohort studies, comparing outcomes for infants receiving and not receiving nirsevimab were included (20–26). In these studies, data were retrieved from health records or specialized databases. Duration of follow-up depended on date of birth (later in the year, shorter follow-up) and ranged from a maximum of two (22) to three (20), four (21, 24, 25) or five (23) months. Participants were a mixture of infants receiving nirsevimab at birth or catch-up. When reported, in most studies, the majority of infants (62%−79%) were receiving nirsevimab at catch-up (20, 22, 24, 27) but, in one study (25), almost all infants received nirsevimab at birth. Average age varied from 3 days to 5 months, depending on the proportion of birth vs. catch-up infants. Coverage rates for nirsevimab varied from 68.7 to 98.6%, with apparently higher rates in at-birth compared with catch-up cohorts where reported.

Nine case-control studies were included (28–36). All studies compared the proportion of infants who had received nirsevimab in cases (with RSV outcome) with the proportion of infants who had received nirsevimab in the control group (non-RSV outcome). In seven studies, the control group consisted of infants admitted or treated for infection (LRTI, ARI or bronchiolitis), who were similar to the case group except that they tested negative for RSV (28, 31–33, 35, 36). In two studies, the control groups were infants admitted to hospital for unrelated causes (29, 30). Studies included infants receiving nirsevimab at birth or catch-up, but none reported the proportions in case or control groups, or for the total population.

### 3.3 Risk of bias assessment

**Figure 2:**
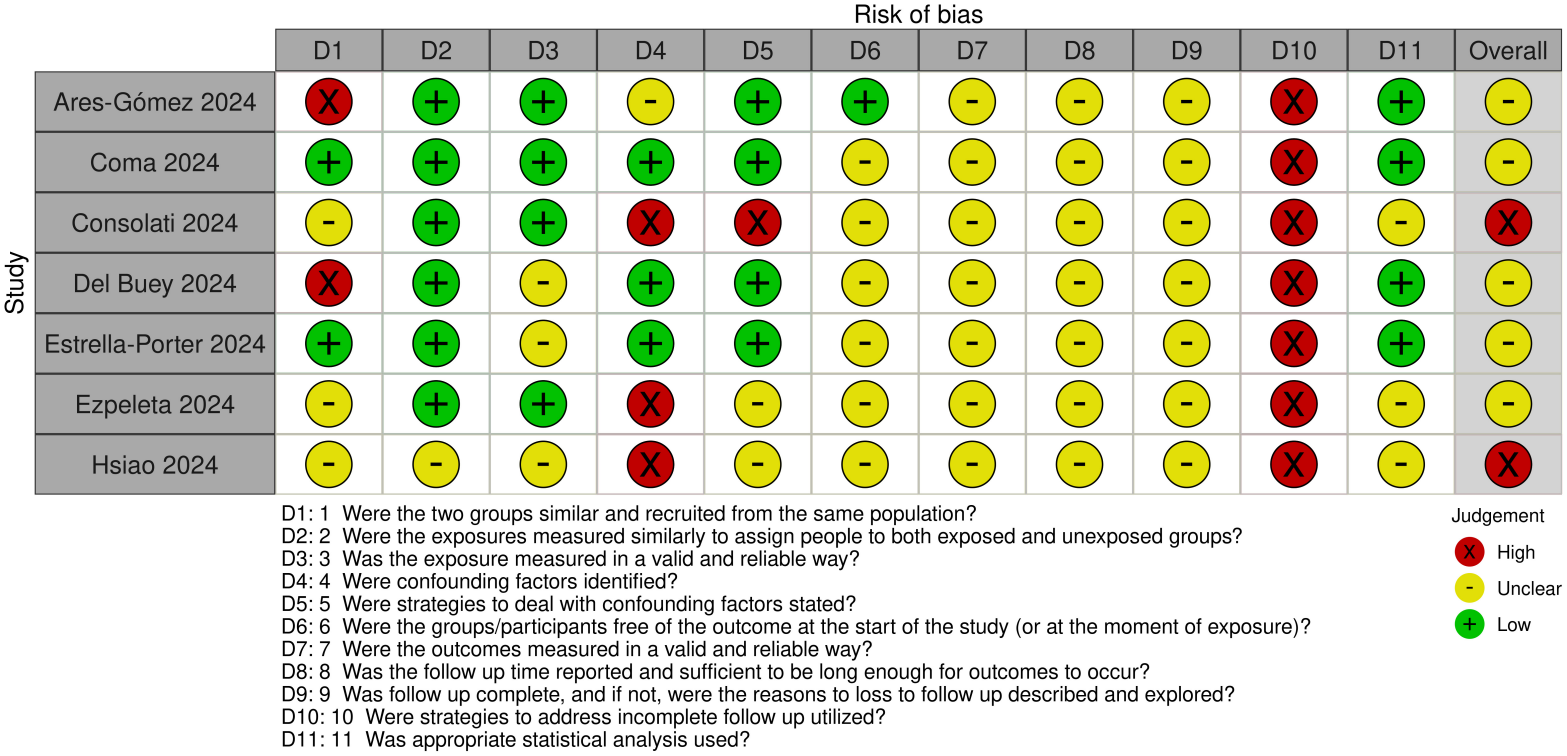
Risk of bias in cohort studies.

**Figure 3:**
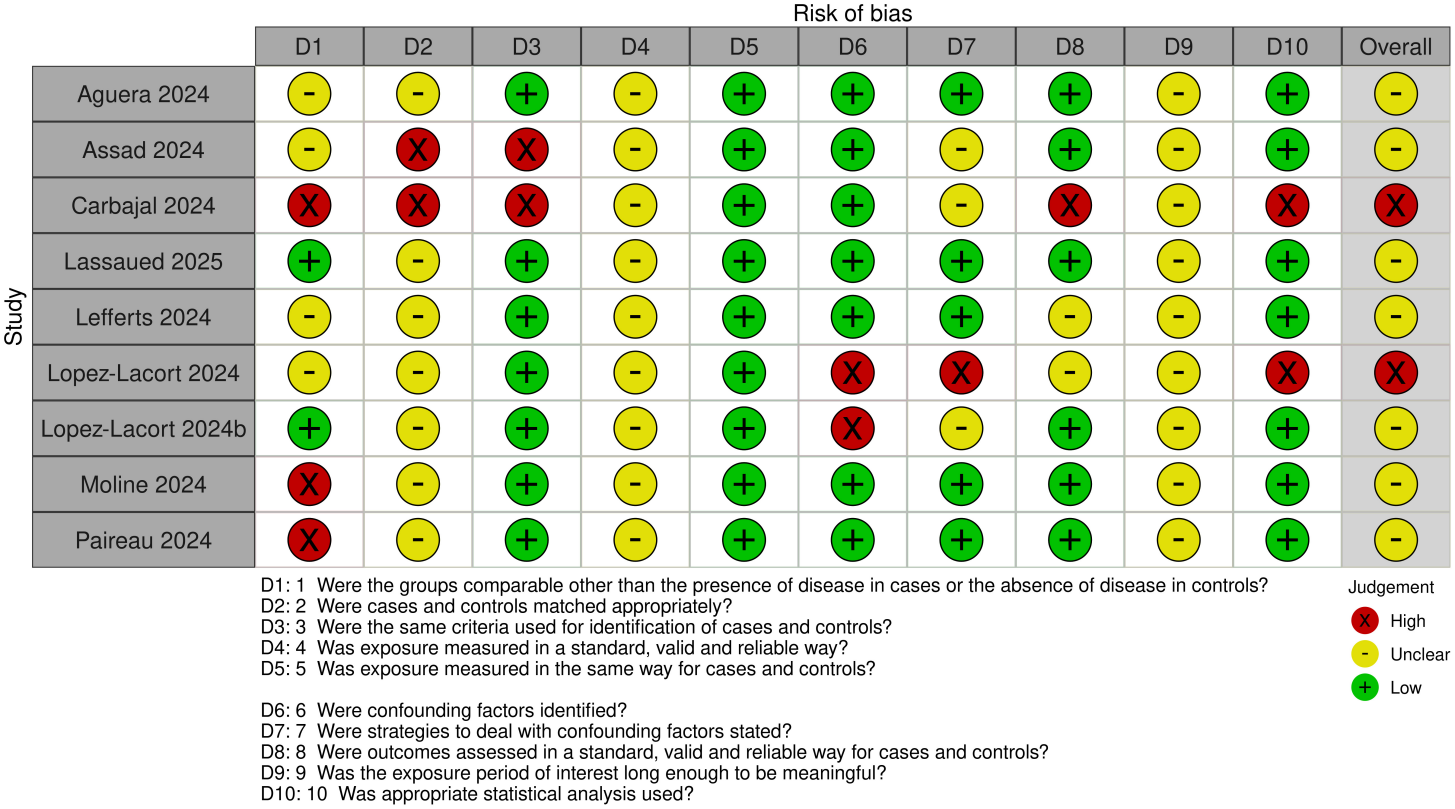
Risk of bias in case-control studies.

Risk of bias assessments for cohort and case-control studies are shown in Figures 2, 3, respectively.

All studies were judged to be at unclear or high risk of bias. The exposure time was short in all studies. Maximum duration of nirsevimab treatment/monitoring with no nirsevimab treatment was 3–4 months. However, minimum duration of treatment/monitoring with no treatment could be less, with some studies including infants with only 1–2 weeks of nirsevimab treatment.

For cohort studies, classification of exposure i.e., whether patients had or had not received nirsevimab treatment, was determined prospectively in most studies (20–22, 25), but classification was done retrospectively (23, 24) or was unclear (26) in other studies. Confounding factors were identified and adjusted for to some extent in most studies, expect in two studies where confounding factors were not identified and limited (25, 26) or no (22) adjustments were made. In most studies, ascertainment of outcome was obtained retrospectively from databases or medical records but, in some studies, the method of ascertainment was unclear. Loss to follow-up was also unclear in all studies and it appears that only infants with complete outcome data were included.

For case-control studies, the comparability of case and control groups was unclear, or differences were noted. Only one study used matching, where it was judged to be insufficient because of the different control population (included infants admitted for non-LRTI related reasons) (29). However, other studies, except for one (33), made adjustments for potential confounders in the analyses. Outcome was assessed prospectively in all studies except one (33) but, in all studies, exposure was ascertained retrospectively from medical records or databases, or the source was unclear.

### 3.4 Outcomes

Nirsevimab showed a statistically significant protective effect for all reported outcomes. The outcomes and estimates of effectiveness are shown in Tables 3– 6. Full event data for all studies is shown in Supplementary material 3.

**Table 3:** Secondary care RSV outcomes.

**Outcome**	***N* studies**	**Study design**	**Sample size**	**Effect measure**	**Effectiveness (%) (95% CI)**
RSV-ARI hospitalization	2 (32, 35)	Case-control	991	aOR	89.8 (77.0–95.5)
RSV-LRTI hospitalization	2 (20, 25)	Cohort studies	11,436	aHR	84.51 (73.7–90.9, *I*^2^: 0%)
	2 (26, 33)	Case control + cohort	32,066	aOR	73.7 (42.3–88.0, *I*^2^: 0%)
	1 (23)	Cohort study	37,067	aHR	Month 1: 93.6 (89.7–96.1) Month 2: 92.5 (89.9–94.4) Month 3: 91.1 (86.9–94.0) Month 4: 89.5 (79.8–94.6) Month 5: 87.6 (67.7–95.3)
RSV-bronchiolitis hospitalization	3 (28–30)	Case-control studies	924^c^	aOR	82.7 (76.4–87.2, *I*^2^: 0%)
	1 (21)	Cohort study	26,525	aHR	87.6 (82.1–91.4)
RSV-LRTI ICU admission	1 (25)	Cohort study	1,177	aHR	85.9 (13.2–97.7)
RSV-bronchiolitis ICU admission	3 (29, 30, 36)	Case-control studies	627^c^	aOR	72.0 (95% CI: 55.3–82.5, *I*^2^: 0%)
	1 (21)	Cohort study	26,525	aHR	90.1 (76.3–95.9)
	1 (23)	Cohort study	37,067	aHR	Month 1: 94.4 (87.3, 97.5) Month 2: 93.3 (85.6, 96.9) Month 3: 92.1 (64.0, 98.3) Month 4: 90.7 (−3.6, 99.2)^a^ Month 5: Not interpretable^b^
RSV-LRTI requiring oxygen support	1 (20)	Cohort study	10,259	aHR	87.1 (70.2, 94.4)
RSV-bronchiolitis requiring ventilation	3 (28–30)	Case-control studies	573^*c*^	aOR	82.6 (−1.5 to 97.0, *I*^2^: 66.5%)

ARI, acute respiratory tract infection; aHR, adjusted hazard ratio; aOR, adjusted odds ratio; ED, emergency department; ICU, intensive care unit; LRTI, lower respiratory tract infection; NR, not reported; RSV, respiratory syncytial virus.

^a^0 events in nirsevimab group.

^b^0 events in both groups.

^c^NR for Carbajal study.

#### 3.4.1 Nirsevimab for preventing hospitalization for RSV-infections

For the prevention RSV-LRTI hospitalization (Table 3), four cohort studies (20, 23, 25, 26) and one case-control study (33) reported significant effectiveness with the use of nirsevimab. Meta-analyses showed different effectiveness estimates: 84.5% (95% CI: 73.7–90.9, *I*^2^: 0%) and 73.7% (42.3–88.0, *I*^2^: 0%), for meta-analysis of studies reporting effectiveness from aHR (adjusted hazard ratios) and aOR (adjusted odds ratios), respectively, with no heterogeneity between studies. However, the CIs of the two estimates overlapped. For the remaining cohort study (23), effectiveness was high in month 1 (93.6%, 95% CI: 89.7–96.1), falling slightly throughout the RSV season, but remaining high at month 5 (87.6%, 95% CI: 67.7–95.3).

For the prevention of RSV-bronchiolitis hospitalization, three case-control studies (28–30) and one cohort study (21) reported nirsevimab effectiveness. Case-control studies showed an effectiveness of 82.7% (95% CI: 76.4–87.2, *I*^2^: 0%), with no observed heterogeneity between studies. The cohort study (21) reported similar effectiveness (87.6%, 95% CI: 82.1–91.4). Similar effectiveness was also observed for the prevention of RSV-ARI hospitalization (89.8%, 95% CI: 77.0–95.5, *I*^2^: 0%) (32, 35).

For the prevention of ICU admission for RSV-bronchiolitis, case control studies (29, 30, 36) showed 72.0% (95% CI: 55.3–82.5, *I*^2^: 0%) effectiveness. However, cohort studies (21, 23) reported higher effectiveness (90%−94%) and, for the prevention of ICU admission for RSV-LRTIs, one cohort study (25) reported 85.9% (95% CI: 13.2–97.7) effectiveness.

Studies also reported the effectiveness of nirsevimab for the prevention of RSV infections requiring ventilatory support. For the prevention of RSV-bronchiolitis requiring ventilation, case-control studies showed an effectiveness of 82.6% (95% Cis: −1.5 to 97.0, *I*^2^: 66.5%). Heterogeneity between studies was high. However, the effectiveness estimate was similar to that reported for the prevention of RSV-LRTI requiring oxygen support (87.1%, 95% CI: 70.2, 94.4).

#### 3.4.2 Nirsevimab for preventing RSV-related primary care attendance

Less evidence was available for the effectiveness of nirsevimab for protecting against milder RSV infections, as observed in primary care settings (Table 4). The effectiveness of nirsevimab for preventing primary care attendance for RSV-ARIs (21, 24), RSV-LRTIs (34) and RSV-bronchiolitis ranged from 69 to 80%. Another study (23) showed a decrease in effectiveness over the RSV season, although RSV cases were not laboratory-confirmed.

**Table 4:** Primary care/outpatient RSV outcomes.

**Outcome**	***N* studies**	**Study design**	**Sample size**	Effect measure^*^	**Effectiveness (%) (95% CI)**
**Primary care RSV outcomes**					
RSV-ARIs in public health centers	1 (24)	Cohort study	27,362	aOR	74 (65–80)
RSV-ARI primary care attendance	1 (21)	Cohort study	26,525	aHR	68.9 (51.7–80)
RSV-LRTI primary care attendance	1 (34)	Case-control	160	aOR	75.8 (40.4–92.7)
RSV-bronchiolitis primary care attendance	1 (24)	Cohort study	37,067	aHR	Month 1: 69.0 (63.5, 73.7) Month 2: 60.9 (55.0, 65.9) Month 3: 50.6 (43.6, 56.7) Month 4: 37.5 (27.6, 46.1) Month 5: 21.1 (5.5, 34.1)
	1 (31) (ambulatory care visits)	Case-control	883	aOR	79.7 (67.7–87.3)
**Medically attended/emergency department attendance**					
Medically attended^a^ RSV-ARIs	1 (32)	Case-control	292	aOR	76 (42–90)
Medically attended^b^ RSV-LRTIs	1 (26)	Cohort study	31,900	aHR	87.2 (81.7–91.1)
RSV-LRTI ED attendance	1 (25)	Cohort study	1,177	aHR	87.9 (70.3–95.1)
RSV-bronchiolitis ED attendance	1 (23)	Cohort study	37,067	aHR	Month 1: 66.7 (61.0, 71.6) Month 2: 58.1 (53.5, 62.3) Month 3: 47.3 (41.2, 52.9) Month 4: 33.8 (21.8, 43.9) Month 5: 16.7 (−5.9, 34.5)
	1 (30)	Case-control	NR	aOR	83 (CI: 71–90)

ARI, acute respiratory tract infection; aHR, adjusted hazard ratio; aOR, adjusted odds ratio; ED, emergency department; ICU, intensive care unit; LRTI, lower respiratory tract infection; NR, not reported; RSV, respiratory syncytial virus.

^a^Hospitalization, emergency department consultation or outpatient clinic visits.

^b^Medical encounters for RSV-LRTIs in any setting.

Other studies reported effectiveness in more general outpatient settings. One cohort study reported 88% effectiveness against RSV-LRTI related ER consultations (25). A case-control study (30) reported 83% effectiveness against RSV-bronchiolitis ED attendance. A cohort study (23), showed lower effectiveness (67%) that decreased throughout the RSV season, although RSV cases were not laboratory-confirmed.

#### 3.4.3 Nirsevimab for preventing non-RSV specific outcomes

For non-RSV specific outcomes (Table 5), single studies reported nirsevimab effectiveness of 43%−69% for the prevention of all-cause hospitalization (20), hospitalization for ARIs (24), LRTIs (20) and bronchiolitis (30). For the prevention of bronchiolitis in primary care (21) and emergency departments (ED) (21, 30), effectiveness of nirsevimab was reported to be 47%−55%.

**Table 5:** Non-RSV specific outcomes.

**Outcome**	***N* studies**	**Study design**	**Sample size**	Effect measure^*^	**Effectiveness (%) (95% CI)**
All cause hospitalization	1 (20)	Cohort study	9,889	aHR	67.7 (58.2–75.1)
ARI-related hospitalization	1 (24)	Cohort study	27,362	OR	42.7 (39.8–45.5)
LRTI-related hospitalization	1 (20)	Cohort study	10,063	aHR	69.3 (56.4–78.4)
Bronchiolitis- related hospitalization	1 (30)	Case-control	2,786	aOR	59 (42–71)
Bronchiolitis-related primary care presentation	1 (21)	Cohort study	26,525	aHR	48.1 (42.4–53.3)
Bronchiolitis-related ED visits	1 (21)	Cohort study	26,525	aHR	55.4 (48.4–61.5)
	1 (30)	Case-control	2,741	aOR	47 (33–58)

ARI, acute respiratory tract infection; aHR, adjusted hazard ratio; aOR, adjusted odds ratio; ED, emergency department; LRTI, lower respiratory tract infection; RSV, respiratory syncytial virus.

#### 3.4.4 Sub-group analysis

Subgroup data reported in full by studies is shown in Supplementary material 2. A summary is shown in Table 6.

**Table 6:** Summary of reported subgroup findings.

**Outcome**	***N* Studies**	**Study design**	**Sample size**	Effect measure^*^	**Effectiveness**
**Birth vs. catch-up**					
All-cause LRTI-hospitalization: at birth	1 (20)	Cohort study	3,340	aIRR	58.7 (15.15–77.47)
All-cause LRTI-hospitalization: catch-up	1 (20)	Cohort study	6,916	aIRR	72.95 (58.41–82.07)
RSV-LRTI primary care attendance: at birth	1 (34)	Case control	32	aOR	NR
RSV-LRTI primary care attendance: catch-up	1 (34)	Case control	128	aOR	80.2 (44.3–95.4)
**Age groups**					
RSV-bronchiolitis related hospitalization: 0–3 months	2 (28–30)	Case-control	NR^b^	aOR	80.1 (71.2–86.2, *I*^2^: 0%)
RSV-bronchiolitis related hospitalization: 3–6 months	2 (28–30)	Case-control	NR^c^	aOR	85.3 (70.8–92.6, *I*^2^: 0%)
RSV-bronchiolitis related hospitalization: >6 months	1 (30)	Case-control	NR	aOR	89 (72–97)
RSV-bronchiolitis ambulatory care attendance: 0–3 months	1 (31)	Case-control	88	aOR	65.5 (−0.8 to 94.0)
RSV-bronchiolitis ambulatory care attendance: 3–6 months	1 (31)	Case-control	290	aOR	87.8 (66.9–95.5)
RSV-bronchiolitis ambulatory care attendance: >6 months	1 (31)	Case-control	505	aOR	82.0 (62.2–91.5)
**Term vs. pre-term**					
RSV-bronchiolitis related hospitalization: term	1 (28)	Case control	NR	aOR	NR
RSV-bronchiolitis related hospitalization: pre-term	1 (28)	Case control	NR	aOR	98.9 (33–100)
RSV-bronchiolitis ambulatory care attendance: term	1 (31)	Case-control	645	aOR	77.7 (62.5–86.8)
RSV-bronchiolitis ambulatory care attendance: pre-term	1 (31)	Case-control	65	aOR	56.6 (−1.2 to 92.5)
**1st vs. 2nd season**					
Medically attended^a^ RSV-ARIs: 1st season (aged < 8 months)	1 (32)	Case-control	292	aOR	76 (42–90)
Medically attended^a^ RSV-ARIs: 2nd season (aged 8–19 months)	1 (32)	Case-control	292	aOR	88 (48–97)

ARI, acute respiratory tract infection; aHR, adjusted hazard ratio; aIRR, adjusted incidence rate ratio; aOR, adjusted odds ratio; LRTI, lower respiratory tract infection; RSV, respiratory syncytial virus.

^a^Hospitalization, emergency department consultation or outpatient clinic visits.

^b^Sample size not reported for Aguera 2024. Sample size for Assad 2024: 482.

^c^Sample size not reported for Aguera 2024. Sample size for Assad 2024: 553.

One study reported subgroup data for seasonal (nirsevimab given at birth) and catch-up cohorts (20). This showed a tendency for higher effectiveness for preventing all cause LRTI-hospitalization for nirsevimab given at catch-up compared with at birth, and similar trends for other outcomes (where effectiveness could not be calculated) were shown (see Supplementary material 2). Another study (34) reported effectiveness for primary care attendance as higher in the catch-up group compared to the total population, but did not report effectiveness for the at-birth subgroup.

Three studies (28–30) reported subgroup data for infants 0–3 vs. 3–6 months. In the meta-analysis of two studies, there was no significant difference in effectiveness for the prevention of RSV-bronchiolitis related hospitalization for infants aged 0–3 compared with 3–6 months. Another study (31) tended to show (non-significant) lower effectiveness for preventing RSV-bronchiolitis ambulatory care attendance in infants aged 0–3 compared with 3–6 and >6 months.

Two studies evaluated nirsevimab in premature infant populations. One study (31) showed a non-significant trend to lower effectiveness for preventing RSV-bronchiolitis ambulatory care attendance for pre-term compared to term infants. However, another study (28) reported high effectiveness for preventing RSV-bronchiolitis hospitalization in premature infants (98.9%; 95% CI: 33–100; *N* = 39), but did not report effectiveness in non-premature infants (*N* = 142).

Finally, in one case-control study (32), there was a non-significant tendency to higher effectiveness for preventing medically attended RSV-ARIs in infants in their first RSV-season compared to infants in their second RSV season.

## 4 Discussion

This review identified observational studies evaluating the effectiveness of nirsevimab when used in the 2023/2024 RSV season. These studies provide “real-world” evidence that nirsevimab is effective for the prevention of RSV-LRTIs. Studies reported a large variety of outcomes and, due to the small number of studies for each outcome, synthesis was more difficult. However, the range of outcomes gives an overview of the likely effect of nirsevimab for preventing different severities of RSV-infection.

Findings appear to be reasonably consistent with published RCTs. In the large HARMONIE RCT (*N* = 8,058) (5), efficacy for the prevention of RSV-LRTI hospitalization was 83.2% (95% CI: 67.8–92.0). Another RCT found lower efficacy for preventing RSV-LRTI hospitalization (62.1%; 95% CI: −8.6 to 86.8) (14). Findings from this review are within this range, with 84.5 (95% CI: 73.6–90.9, *I*^2^: 0%) and 73.7 (42.3–88.0, *I*^2^: 0%) from the meta-analyses of cohort studies and case-control studies, respectively in this review.

Since this review did not include other monoclonal antibodies, it is not possible to conclude the effectiveness of nirsevimab compared with agents such as palivizumab and clesrovimab. Indeed, there are no head-to-head efficacy studies comparing nirsevimab with these monoclonal antibodies [although one compares safety with palivizumab (37)]. However, from RCT data, it appears that nirsevimab is likely to have comparable or greater efficacy compared to these agents. Clesrovimab demonstrated 60% effectiveness for the prevention of medically attended RSV-LRTIs in an RCT of healthy term or pre-term infants (38). In high-risk infants and children, palivizumab shows a risk ratio of 0.44 for RSV-LRTI hospitalization (39), equating to effectiveness of 56%. Some palivizumab trials have longer follow-up compared with nirsevimab studies (up to 2 years compared with seasonal assessment). However, if it is assumed that nirsevimab would be given seasonally to high-risk infants/children, these rates may be compared, showing nirsevimab to have at least comparable effectiveness.

As would be anticipated, nirsevimab showed lower effectiveness for non-RSV specific outcomes. Since RSV is one of several pathogens causing LTRIs and other ARIs, and nirsevimab is an agent that specifically targets RSV, effectiveness for the prevention of all-cause infection is lower. However, it can be noted that nirsevimab showed significant, high effectiveness for the prevention of all-cause hospitalization (67.7%) and LRTI-hospitalization (59%−69.3%). This may be due to the high proportion of LTRIs attributable to RSV.

Although data are limited for comparisons across different studies, findings appear to show that nirsevimab may have had less effectiveness for preventing RSV infections presenting in primary care. For example, effectiveness for preventing hospitalization or ICU admission due RSV-LRTIs or RSV-bronchiolitis tended to be higher than those for primary care RSV-LRTIs or RSV-bronchiolitis. However, this may be due to difference in study methods. Hospital settings tended to use PCR confirmation for RSV whereas, in some primary care studies, “suspected” cases of RSV were included. Inaccuracies in RSV confirmation may have led to underestimation of true effectiveness in these settings. If the apparent positive rate is uniformly increased in both nirsevimab and control groups, the relative difference, and therefore apparent effectiveness, is reduced. Therefore, the effectiveness of nirsevimab for preventing milder/primary care presenting RSV, may be higher than that indicated in this review.

Some studies examined whether nirsevimab has different effectiveness when used at birth compared to at catch-up. One study (20) showed higher rates of effectiveness for preventing all cause LRTI hospitalization in the catch-up group. Effectiveness was not reported for the at birth group for RSV-LRTI hospitalization or all-cause hospitalization, but adjusted IRRs were higher for the at birth cohort. In another study (34) only the catch-up, and not at-birth, subgroup was reported, but the catch-up group effectiveness was higher than the overall population effectiveness.

The evidence appears to be mixed regarding the relative effectiveness of nirsevimab when given at birth vs. catch-up. In the sub-group meta-analysis, lower rates of effectiveness tended to be observed for infants aged 0–3 compared with 3–6 months, and infants given nirsevimab at birth may have comprised a reasonable proportion of this group. However, the study by Ezpeleta et al. (25), where nirsevimab was primarily given at birth (96% of participants), showed effectiveness for the prevention of RSV-LRTI hospitalization equivalent to studies in mixed populations. Additionally, in the HARMONIE RCT (5), in France, the location where the highest efficacy for the prevention of RSV-LRTI hospitalization was reported (89.6%; 95% CI: 58.8–98.7), the majority of participants were born during the RSV season (67%), presumably receiving nirsevimab at birth. The impact of administration at birth vs. at catch-up is therefore still unclear and it is not possible to determine whether effectiveness varies in these groups.

The effectiveness of nirsevimab in term vs. preterm infants was evaluated in one study (31), showing higher effectiveness in the term subgroup. However, another study showed high effectiveness (98%) in pre-term infants (28). Findings from an RCT in pre-term infants (10) showed similar efficacy for the prevention of RSV-LRTI hospitalization compared with studies in general infant populations. Therefore, there does not currently appear to be clear evidence about the relative effectiveness of nirsevimab in term and pre-term infants.

### 4.1 Limitations of the evidence

Observational evidence is limited due to the uncontrolled nature of studies; confounding being a major issue in their interpretation. In this review, the majority of included cohort and case control studies made adjustments for potential confounders. Important confounders were identified to be age and high-risk status. However, in cohort studies reporting baseline characteristics, there do not appear to have been marked differences. Findings were reasonably consistent with RCT evidence. Although this does not prove that studies were unbiased, it gives some assurance that results are useful in corroborating findings from controlled settings.

Another limitation of the included studies was that infant follow-up did not extend over the whole RSV season. Unavoidably, most infants were born within the observation period, resulting in a period of evaluation shorter than the full RSV season for most studies. Nirsevimab has claimed efficacy over a 6-month RSV season (16) and longer follow-up periods would be required to test this in observational studies. Because the effectiveness of nirsevimab is likely to reduce over time, this may have artificially increased the apparent whole season effectiveness. However, in a study that examined effectiveness by month (23), although it fell during the RSV season, it still remained high, for example RSV-LRTI hospitalization was 89.5 and 87.6% in months 4 and 5, respectively.

A final limitation was the wide range of outcomes reported, making it difficult to conduct meta-analyses and examine the consistency of findings across studies. However, reporting of the range of outcomes is useful for identifying potential trends in the types of outcomes showing greater effectiveness. For example, in the observational studies, nirsevimab appeared to show greater effectiveness for outcomes related to severe disease, but this would need to be confirmed with more evidence.

### 4.2 Future research

Future research could include well planned observational studies, such as prospective cohort studies, to examine nirsevimab effectiveness. For new studies, it would be useful to have better consistency in outcomes e.g., to consistently report RSV-LRTI outcomes. This would allow comparison of findings across studies and more firm conclusions to the drawn. More detailed information and control of the study period would help to give assurance that nirsevimab is effective throughout the whole RSV season. For example, subgroup data for infants given nirsevimab at the start of the season and followed up for its duration could be reported. Finally, as most RSV LRTIs are seen in primary care, more research of the effectiveness of nirsevimab in primary care settings is needed. Studies should use laboratory confirmation where possible.

### 4.3 Conclusions

Real-word evidence suggests that nirsevimab is effective for the prevention of RSV-LRTI-related outcomes, such as RSV-LRTI hospitalization and ICU admission. There is no clear evidence that effectiveness differs for at birth compared with catch-up groups, or that effectiveness varies in preterm infants compared to general infant populations.

## Data Availability

The original contributions presented in the study are included in the article/Supplementary material, further inquiries can be directed to the corresponding author.
