# Supplementary material for: Nirsevimab for preventing respiratory syncytial virus lower respiratory tract infections in infants: a systematic review and meta-analysis
Source: Front Public Health. 2025 Oct 24;13:1641085. doi: 10.3389/fpubh.2025.1641085 (PMC12592186; doi:10.3389/fpubh.2025.1641085)
Supplement: Supplementary file 1 [file Data_Sheet_1.DOCX]

Supplementary material 1: Study Protocol

Nirsevimab for Preventing Respiratory Syncytial Virus Lower Respiratory Tract Infections in Neonates and Infants

Review Protocol

RACHAEL MCCOOL, Associate Director

MARY CHAPPELL, Senior Research Consultant

ERIN BARKER, Senior Research Consultant

PAUL MILLER, Information Specialist

KATIE REDDISH, Research Assistant

25/07/2024

[Abbreviations 3](#_Toc171953143)

[1 Background 4](#_Toc171953144)

[1.1 Objectives 4](#_Toc171953145)

[2 Eligibility Criteria 5](#_Toc171953146)

[2.1 Protocol Amendments 6](#_Toc171953147)

[3 Methods 6](#_Toc171953148)

[3.1 Identifying Relevant Studies 6](#_Toc171953149)

[3.1.1 Search strategy 6](#_Toc171953150)

[3.2 Resources to be Searched 7](#_Toc171953151)

[3.2.1 Running the search strategies and downloading results 9](#_Toc171953152)

[3.3 Study Selection 9](#_Toc171953153)

[3.4 Mapping 10](#_Toc171953154)

[3.5 Data Extraction 10](#_Toc171953155)

[3.6 Risk of Bias 12](#_Toc171953156)

[3.7 Synthesis and Analysis 12](#_Toc171953157)

[3.7.1 Feasibility assessment and analysis plan 12](#_Toc171953158)

[3.7.2 Meta-analysis 13](#_Toc171953159)

[3.7.3 Subgroup/sensitivity analysis 13](#_Toc171953160)

[3.8 Reporting 13](#_Toc171953161)

[4 Deliverables and Timelines 14](#_Toc171953162)

[4.1 Deliverables 14](#_Toc171953163)

[4.2 Timeline 14](#_Toc171953164)

[5 References 16](#_Toc171953165)

[6 Appendix A – PRISMA Record Selection Process 19](#_Toc171953166)

[7 Appendix B – PRISMA Checklist 20](#_Toc171953167)

[8 Appendix C – Protocol Amendments 23](#_Toc171953168)

All reasonable precautions have been taken by YHEC to verify the information contained in this publication. However, the published material is being distributed without warranty of any kind, either expressed or implied. The responsibility for the interpretation and use of the material lies with the reader. In no event shall YHEC be liable for damages arising from its use. York Health Economics Consortium is a Limited Company. Registered in England and Wales No. 4144762. Registered office as shown.

Abbreviations

CADTH Canadian Agency for Drugs and Technologies in Health

CDSR Cochrane Database of Systematic Reviews

CENTRAL Cochrane Central Register of Controlled Trials

CI Confidence interval

CPCI-S Conference Proceedings Citation Index – Science

EMA European Medicines Agency

FDA Food and Drug Administration

HTA Health Technology Assessment

ICER Institute for Clinical and Economic Review

ICTRP WHO International Clinical Trials Registry Platform

IJCME International Committee of Medical Journal Editors

GPP3 Good Publication Practice for Company Sponsored

LTRI Lower respiratory tract infection

NICE National Institute for Health and Care Excellence

NMA Network meta-analysis

OR Odds ratio

PA Protocol amendment

RCT Randomised controlled trial

RR Relative risk

RSV Respiratory syncytial virus

RWE Real-world evidence

WMD Weighted mean difference

YHEC York Health Economic Consortium

# Background

Respiratory syncytial virus (RSV) is a common virus, causing cold-like symptoms in young children. However, in some of these cases, RSVs can cause lower respiratory tract infections (LRTIs) that can lead to hospitalisation or death [1].

Nirsevimab has been developed jointly by Sanofi and AstraZeneca for the prevention of RSV LRTIs in neonates and infants [2] and is approved in this indication in several countries including the UK, EU and USA for the infant’s first RSV season and second season for the most high risk group [2, 3]. It is administered before the RSV season, or at birth during the season, as a single 50 or 100mg dose (depending on body weight) to cover a complete RSV season [4].

Several RCTs have been conducted to evaluate nirsevimab for the prevention of RSV LRTIs in neonates and infants [5-7]. There is also recent real-world evidence of its effectiveness in reducing hospital admission for RSV LRTIs and related outcomes in cross sectional [8-14], cohort [15, 16] and case control [17-19] studies. This data provides opportunity to gain an overall view of the effectiveness of nirsevimab in real-world practice.

## Objectives

The objective of this work is to conduct a systematic review and meta-analysis of real-world studies of the effectiveness of nirsevimab in reducing RSV LRTIs.

# Eligibility Criteria

This systematic review will be undertaken according to the principles of systematic reviewing embodied in the Cochrane handbook [20] and guidance published by the Centre for Reviews and Dissemination (CRD) [21]. This protocol will be registered on the PROSPERO database [22].

The eligibility criteria are summarised in Table 2.1.

Table 2.1: Eligibility criteria

|  | Inclusion criteria | Exclusion criteria |
| --- | --- | --- |
| Population | - Neonates (under 28 days) and infants (under 1 year) in their first RSV season. - High risk children in their second RSV season. | - Older children and adults. - Infants not in their first RSV season. |
| Intervention | - Nirsevimab. | - Other intervention. |
| Comparators | - Any treatment. - No comparator. | - None. |
| Outcomes | Relevant outcomes, including:   - Nirsevimab coverage. - Hospitalisation due to RSV LRTIs. - Emergency room visit associated with RSV. - Primary care visit related to RSV. - Rates of medically attended RSV LRTI. - Rates of ICU medically attended RSV LRTI. - Rates of mechanical ventilation related to RSV. - All cause medically attended LRTI. - All cause LRTI hospitalisation. - Adverse events. | - TBC following study mapping. |
| Study design | - Cohort studies. - Cross sectional studies. - Case-control studies. | - RCTs. - Non-randomised comparative trials. - Case reports. - Narrative reviews. - Systematic reviews.* |
| Limits | - Any language. - Conference abstracts from 2021. | - News articles. - Editorials. - Conference abstracts published prior to 2021 - Preprints. |

Key: LRTI – lower respiratory tract infection; RCT – randomised controlled trial; RSV - Respiratory syncytial virus.

* Relevant systematic reviews published in the previous 3 years will be retained and included studies lists screened to identify additional eligible studies.

Where studies are mixed populations of eligible and ineligible people, if ≥80% of the population meet eligibility criteria, a study may be included. However, this does not apply to intervention coverage. For example:

- A cross-sectional study with 75% coverage in the nirsevimab group reporting outcomes for the whole eligible population would be included,
- But a study reporting outcome in the nirsevimab group where 30% of people received nirsevimab in their second RSV season would be excluded.

However, studies excluded on the basis of being mixed populations of eligible and ineligible people will be tagged, so that they can be retrieved if needed at a later date.

## Protocol Amendments

Any essential protocol amendments or clarifications will be recorded in Appendix C. Changes will be made to the text of the protocol and flagged with [PA#].

# Methods

## Identifying Relevant Studies

### Search strategy

A MEDLINE (OvidSP) search strategy designed to identify studies of nirsevimab is presented in Figure 3.1.

The strategy comprises terms for nirsevimab only. Given the low number of records retrieved for nirsevimab, it is not necessary to include search terms for RSV. Adding population terms could result in potentially relevant records being missed by the search.

The strategy was devised using a combination of subject indexing terms and free text search terms in the Title, Abstract and Keyword Heading Word fields. The search terms were identified through discussion within the research team and browsing database thesauri.

The strategy excludes animal studies from MEDLINE using a standard algorithm (search line 4). The strategy also excludes some ineligible publication types which are unlikely to yield relevant study reports (editorials and news items) (search line 5).

Reflecting the eligibility criteria, the strategy is not restricted by date or language.

The final Ovid MEDLINE strategy will be peer-reviewed before execution by a second Information Specialist. Peer review will consider the appropriateness of the strategy for the review scope and eligibility criteria, inclusion of key search terms, errors in spelling, syntax and line combinations, and application of exclusions.

Figure 3.1: Search strategy for Ovid MEDLINE® ALL

1 nirsevimab*.ti,ab,kf,rn,nm,ot. 152

2 (beyfortus*2 or nirsevimab or MEDI8897*2 or medi 8897*2 or 1989556-22-0 or VRN8S9CW5V or MED 18897*2 or MED18897*2 or sp 0232*2 or sp 232*2 or sp0232*2 or sp232*2).ti,ab,kf,rn,nm,ot. 81

3 or/1-2 169

4 exp animals/ not humans/ 5241653

5 (news or editorial).pt. 925488

6 or/4-5 6139531

7 3 not 6 159

Key to Ovid symbols and commands:

* Unlimited right-hand truncation symbol

*N Limited right-hand truncation - restricts the number of characters following the word to N

ti,ab,kf,rn,nm,ot Searches are restricted to the Title (ti), Abstract (ab), Keyword Heading Word (kf), Registry Number/Name of Substance (rn), Name of Substance Word (nm) and Original Title (ot) fields

/ Searches are restricted to the Subject Heading field

exp The subject heading is exploded

pt. Search is restricted to the publication type field

or/1-2 Combines sets 1 to 2 using OR

Saved in Ovid as: temp - Sanofi nirsevimab - medline - for protocol

## Resources to be Searched

We will conduct the literature search in the databases and information sources shown in Table 3.1.

Table 3.1: Databases and information sources to be searched

| Resource | Interface / URL |
| --- | --- |
| Databases | |
| MEDLINE(R) ALL | OvidSP |
| Embase | OvidSP |
| Cochrane Database of Systematic Reviews (CDSR) | Cochrane Library/Wiley |
| Cochrane Central Register of Controlled Trials (CENTRAL) | Cochrane Library/Wiley |
| HTA Database | https://database.inahta.org/ |
| Conference Proceedings Citation Index – Science (CPCI-S) | Web of Science |
|  |  |
| Trials Registers | |
| ClinicalTrials.gov | https://clinicaltrials.gov/ |
| WHO International Clinical Trials Registry Platform (ICTRP) | https://trialsearch.who.int/ |
|  |  |
| HTA / regulatory agency webpages | |
| Drugs@FDA (Food and Drug Administration) | https://www.accessdata.fda.gov/scripts/cder/daf/index.cfm |
| Centers for Disease Control and Prevention (CDC) Advisory Committee on Immunization Practices (ACIP) | https://stacks.cdc.gov/advancesearch |
| European Medicines Agency (EMA) medicines webpages | https://www.ema.europa.eu/en |
| National Institute for Health and Care Excellence (NICE) webpages | https://www.nice.org.uk/ |
| Canadian Agency for Drugs and Technologies in Health (CADTH) webpages | https://www.cadth.ca/ |
| Institute for Clinical and Economic Review (ICER) webpages | https://icer-review.org/ |
|  |  |
| **Non-database conference searches** | As detailed below. |
|  |  |
| **Reference list checking** | n/a |

The trials register sources listed above (ClinicalTrials.gov and ICTRP) will be searched to identify information on studies in progress. A number of data providers provide data to WHO for inclusion in ICTRP, including the EU Clinical Trials Register (EU-CTR).

In addition to searching the HTA database, targeted searches of the listed technology assessment and regulatory agency websites will be conducted:

- Drugs@FDA for medical, statistical and other reviews.
- Centers for Disease Control and Prevention (CDC) Advisory Committee on Immunization Practices (ACIP) for scientific data presented at ACIP meetings (2023 onwards).
- European Medicines Agency (EMA) medicines webpages for European public assessment reports (EPARs).
- National Institute for Health and Care Excellence (NICE) webpages for company submissions to NICE, Final Appraisal Determination documents, Evidence Review Group (ERG) reports (for single technology assessments only), and assessment reports (for multiple technology appraisals only).
- Canadian Agency for Drugs and Technologies in Health (CADTH) webpages for CADTH Clinical Guidance, and CADTH Final Recommendations associated with Reimbursement Reviews.
- Institute for Clinical and Economic Review (ICER) webpages for Final Evidence Reports (or Draft Evidence Reports if final is not yet available) and Evidence Presentation (if available).

Reflecting the eligibility criteria, CPCI-S search results and records indexed in Embase as conference abstracts will be restricted to studies published from 2021 to date. Reflecting the eligibility criteria, records that are indexed as preprints will be excluded from Embase search results.

Recent research published as conference abstracts will be identified by searching Embase (which indexes a significant number of conference publications) and CPCI-S (a conference proceedings citation index for science disciplines). The following conferences have been identified as highly relevant. We will check Embase and CPCI-S to ascertain if they include records for these conferences for the time period required (conferences held between 2021 and the date of the search). If records for the conference of interest are found in Embase or CPCI-S, an assumption is made that the conference is fully indexed in the database, and that no searches outside these resources are required. If records for the required conferences are not included in Embase or CPCI-S, YHEC will undertake searches of proceedings via conference webpages or journal supplements, if these are available online free of charge.

- ISPOR EU
- ISPOR US
- European Society for Paediatric Infectious Diseases (ESPID) Annual Meeting
- ReSViNET Conference

We will also check included studies list of any retrieved relevant systematic reviews published in the last three years for any eligible studies that may have been missed by the database searches.

For each paper that is selected for inclusion in the review, a check will also be made to establish if any of the following notices are associated with the included paper: retraction notice, erratum notice, corrected and republished paper notice, expression of concern notice. The check will be conducted via the PubMed record for the paper or (if no PubMed record is found) via the journal webpage for the paper. If a relevant notice is associated with an included paper, the notice will be assessed by the review team.

### Running the search strategies and downloading results

We will conduct searches using each database or resource listed in the protocol, translating the agreed Ovid MEDLINE strategy appropriately. Translation includes consideration of differences in database interfaces and functionality, in addition to variation in indexing languages and thesauri. The final translated database strategies will be peer-reviewed by a second Information Specialist. Peer review will consider the appropriateness of the translation for the database being searched, errors in syntax and line combinations, and application of exclusions.

We will document all search strategies and search results and we will provide this in the final report to meet standard requirements for clear formal reporting of the search process. The report of search methods will be informed by the PRISMA-S (Preferred Reporting Items for Systematic reviews and Meta-Analyses literature search extension) checklist [23] and the PRISMA 2020 statement [24, 25].

Where possible, we will download the results of searches in a tagged format and load them into bibliographic management software (EndNote) [26]. The results will be deduplicated using several algorithms and the deduplicated references held in a duplicates EndNote database for checking if required. Results from resources which do not allow export in a format compatible with EndNote will be saved in Word or Excel documents as appropriate and manually deduplicated.

## Study Selection

Study selection will be undertaken as follows:

- The titles and abstracts of remaining records will be assessed for relevance by double independent reviewer selection with disagreements adjudicated by a third reviewer.
- We will obtain the full text of potentially relevant studies and these will be assessed for relevance by double independent reviewer with disagreements adjudicated by a third reviewer.

We will record the number of records included and removed at each stage in the PRISMA flow diagram. Studies excluded after assessment of the full document for each review will be described in an excluded studies table with the reasons for exclusion.

We will obtain electronic or paper copies of potentially relevant full documents meeting the systematic review’s eligibility criteria in liaison with Sanofi or via local access routes.

We will share the final selection of studies with Sanofi and we will discuss any issues arising before proceeding to data extraction.

Where results for one trial/study are reported in more than one paper, all related papers will be identified and grouped together to ensure that participants in individual trials/studies are only included once.

## Mapping

Following study selection, studies meeting eligibility criteria will be mapped in an Excel spreadsheet to provide brief study details and identify common outcomes. The mapping will include:

- Study design.
- Country of origin.
- Sample size.
- Reported outcomes, along with statistical measure e.g. risk ratio or odds ratio.
- Outcome data for the effectiveness of nirsevimab for reducing RSV LRTI-related hospitalisations. This data will be used in an analysis for the ID Week abstract.

The results of mapping will be discussed with Sanofi to determine the outcomes to take forward to meta-analysis. On this basis, studies will be selected for data extraction according to the outcomes that they report.

## Data Extraction

For studies selected to move forward to data extraction, data will be extracted into a YHEC template in Excel. We will ensure that any information required to inform the similarity assessment and meta-analysis is extracted in a format that can feed directly into the required output to minimise the need for further data manipulation and the risk of introducing errors.

A single reviewer will extract the data into an Excel template and every data point will be checked by a second reviewer. Disagreements will be adjudicated by a third reviewer.

We will extract the following elements from the eligible studies:

- Trial/study details (bibliographic details).
- Trial/study characteristics:
- Study design.
- Study objective.
- Number of participating centres and countries.
- Eligibility criteria, including eligibility for palivizumab.
- Number of patients analysed.
- Length of RSV season assessed.
- Follow up duration.
- Case definition i.e. methods for confirmation of RVS LRTI positive cases.
- Patient baseline characteristics:
- Age.
- Gender.
- Proportion preterm birth (with definition of preterm).
- Proportion of infants with comorbidities if available.
- Weight at birth.
- Details of intervention and comparator
- Treatment (if any).
- Nature of the control group.
- Date of data collection.
- Details of statistical analyses.
- For each of the outcomes specified we will extract the following:
- Outcome definition.
- The unit of measurement.
- The number of patients included in the analysis.
- The size of the effect:
  - - For dichotomous outcomes; absolute and relative risks (or odds ratios) and risk (or rate) differences.
    - For continuous outcomes; the mean change and measure of variance from baseline (or at both baseline and final visit), or mean difference between treatments.
    - For time-to-event analysis; the number of events in each arm, median time to event and a hazard ratio and p-value.
    - Where possible, absolute and relative data will be extracted.
- A measure of precision for each estimate of effect (95% confidence intervals, standard error or standard deviation).

Where reported, we will extract data for the following subgroups separately:

- Pre-term versus healthy term infants.
- Infants eligible for palivizumab versus infants ineligible for palivizumab.
- Infants and neonates receiving nirsevimab at birth versus at catch up.

We will not collect data for other reported subgroups or sensitivity analyses. We will extract outcomes at all reported timepoints.

## Risk of Bias

The risk of bias assessment will be carried out using tools appropriate to the included study designs, and will be taken into account when considering the results of the studies. The risk of bias assessment will be carried out alongside data extraction by a single reviewer with every assessment checked by a second reviewer.

We will summarise the results of the risk of bias assessment in a table and we will provide a detailed assessment in an Appendix to the main report.

Table 3.2 lists the risk of bias assessment tools that we will use for each type of study design.

Table 3.2: Risk of bias assessment tools

| Study design | Tool to use |
| --- | --- |
| Case control studies | Joanna Briggs Institute (JBI) Checklist for Case Control Studies [27] |
| Cohort Studies  (group of patients with two or more study interventions followed over time) | JBI Checklist for Cohort Studies [27] |
| Cross-sectional studies | JBI Checklist for Analytical Cross Sectional Studies [27] |

Key: JBI - Joanna Briggs Institute

## Synthesis and Analysis

### Feasibility assessment and analysis plan

We will assess the similarity of studies and availability of data, and present this in PowerPoint slides. These will present the potential outcomes for analyses and any issues associated with the inclusion of studies in these analyses. We will provide these slides to Sanofi to aid discussion on the suitability and composition of meta-analysis.

Based on the findings of the feasibility assessment, we will discuss the most appropriate analyses to conduct with Sanofi. We will confirm what outcomes will be analysed, and what studies will contribute to each meta-analysis. We will agree a plan for how we will report the results.

### Meta-analysis

Where studies are similar enough and data are available, we will undertake statistical pooling, in the form of meta-analysis using R. Where meta-analysis is possible (i.e. where there is statistical and clinical homogeneity), we will pool data using both fixed- and random-effects models. We will provide forest plots of the results. Up to 15 distinct meta-analyses will be conducted based on the outcomes of interest, time points, subgroups and/or sensitivity analyses.

We will assess heterogeneity both by visual inspection of the forest plots and by a formal statistical test for heterogeneity using a Chi² test on N-1 degrees of freedom, with an alpha of 0.05 used for statistical significance and with the I² test. I² values of 25%, 50% and 75% correspond to low, medium and high levels of heterogeneity.

Where appropriate (i.e. in meta-analyses with more than 10 trials similar in terms of their patient and study characteristics) we will explore potential publication bias by generating a funnel plot and statistically testing using a linear regression test. If there are fewer than ten studies, the power of the test is usually deemed too low to distinguish chance from true asymmetry [28].

### Subgroup/sensitivity analysis

Subject to available data, subgroup and/or sensitivity analyses will be conducted. These will be decided in discussion with Sanofi following the feasibility assessment, before data extraction begins. For, example we may conduct the following subgroup analyses:

- Pre-term versus healthy term infants.
- Infants eligible for palivizumab versus infants ineligible for palivizumab.
- Infants and neonates receiving nirsevimab at birth versus at catch up.

## Reporting

The review will be reported in a set of PowerPoint slides, summarising the methods, findings and outcome data. This will include a summary of all methods, a study flow diagram, study characteristics tables for included studies and results, including forest plots and tables of outcome data.

The full search strategies and a table of studies excluded at full-text assessment will also be provided.

# Deliverables

## Deliverables

The following documents will be provided during the project:

- Draft and final review protocol.
- Included studies list following study selection.
- Outcome mapping in Excel.
- Draft and final report in PowerPoint slides, including:
- List of included studies.
- Completed PRISMA flow diagram.
- Forest plots.
- Additional documents:
- Full search strategies.
- List of excluded studies with reasons for exclusion.
- Draft and final abstract for conference submission.
- Drafts and final version of a manuscript.

# References

1. Shi T, McAllister DA, O'Brien KL, Simoes EAF, Madhi SA, Gessner BD*, et al.* Global, regional, and national disease burden estimates of acute lower respiratory infections due to respiratory syncytial virus in young children in 2015: a systematic review and modelling study. Lancet. 2017.390(10098):946-58. doi: 10.1016/S0140-6736(17)30938-8

2. Keam SJ. Nirsevimab: first approval. Drugs. 2023.83(2):181-87. doi: https://dx.doi.org/10.1007/s40265-022-01829-6

3. Sevilla JP. Immunization, not vaccination: monoclonal antibodies for infant RSV prevention and the US vaccines for children program. J Med Econ. 2023.26(1):991-97. doi: https://dx.doi.org/10.1080/13696998.2023.2242169

4. European Medicines Agency. Nirsevimab (Beyfortus): summary of product characteristics. 2024. [cited 18 June 2024]. Available from: https://www.ema.europa.eu/en/glossary/summary-product-characteristics.

5. Drysdale SB, Cathie K, Flamein F, Knuf M, Collins AM, Hill HC*, et al.* Nirsevimab for prevention of hospitalizations due to RSV in infants. N Engl J Med. 2023.389(26):2425-35. doi: https://dx.doi.org/10.1056/NEJMoa2309189

6. Griffin MP, Yuan Y, Takas T, Domachowske JB, Madhi SA, Manzoni P*, et al.* Single-dose nirsevimab for prevention of RSV in preterm infants. N Engl J Med. 2020.383(5):415-25. doi: https://dx.doi.org/10.1056/NEJMoa1913556

7. Hammitt LL, Dagan R, Yuan Y, Baca Cots M, Bosheva M, Madhi SA*, et al.* Nirsevimab for prevention of RSV in healthy late-preterm and term infants. N Engl J Med. 2022.386(9):837-46. doi: https://dx.doi.org/10.1056/NEJMoa2110275

8. Ares-Gomez S, Mallah N, Santiago-Perez M-I, Pardo-Seco J, Perez-Martinez O, Otero-Barros M-T*, et al.* Effectiveness and impact of universal prophylaxis with nirsevimab in infants against hospitalisation for respiratory syncytial virus in Galicia, Spain: initial results of a population-based longitudinal study. Lancet Infect Dis. 2024.30:30. doi: https://dx.doi.org/10.1016/S1473-3099(24)00215-9

9. Consolati A, Farinelli M, Serravalle P, Rollandin C, Apprato L, Esposito S, Bongiorno S. Safety and efficacy of nirsevimab in a universal prevention program of respiratory syncytial virus bronchiolitis in newborns and infants in the first year of life in the Valle d'Aosta Region, Italy, in the 2023-2024 epidemic season. Vaccines (Basel). 2024.12(5):17. doi: https://dx.doi.org/10.3390/vaccines12050549

10. Ernst C, Bejko D, Gaasch L, Hannelas E, Kahn I, Pierron C*, et al.* Impact of nirsevimab prophylaxis on paediatric respiratory syncytial virus (RSV)-related hospitalisations during the initial 2023/24 season in Luxembourg. Euro Surveill. 2024.29(4)doi: https://dx.doi.org/10.2807/1560-7917.ES.2024.29.4.2400033

11. Levy C, Werner A, Rybak A, Bechet S, Batard C, Hassid F*, et al.* Early impact of nirsevimab on ambulatory all-cause bronchiolitis: a prospective multicentric surveillance study in France. Journal of the Pediatric Infectious Diseases Societ. 2024.23:23. doi: https://dx.doi.org/10.1093/jpids/piae051

12. Lopez-Lacort M, Munoz-Quiles C, Mira-Iglesias A, Lopez-Labrador FX, Mengual-Chulia B, Fernandez-Garcia C*, et al.* Early estimates of nirsevimab immunoprophylaxis effectiveness against hospital admission for respiratory syncytial virus lower respiratory tract infections in infants, Spain, October 2023 to January 2024. Euro Surveill. 2024.29(6)doi: https://dx.doi.org/10.2807/1560-7917.ES.2024.29.6.2400046

13. Mazagatos C, Mendioroz J, Rumayor MB, Gallardo Garcia V, Alvarez Rio V, Cebollada Gracia AD*, et al.* Estimated impact of nirsevimab on the incidence of respiratory syncytial virus infections requiring hospital admission in children < 1 year, weeks 40, 2023, to 8, 2024, Spain. Influenza other respi. 2024.18(5):e13294. doi: https://dx.doi.org/10.1111/irv.13294

14. Moline HL, Tannis A, Toepfer AP, Williams JV, Boom JA, Englund JA*, et al.* Early estimate of nirsevimab effectiveness for prevention of respiratory syncytial virus-associated hospitalization among infants entering their first respiratory syncytial virus season - New Vaccine Surveillance Network, October 2023-February 2024. MMWR Morb Mortal Wkly Rep. 2024.73(9):209-14. doi: https://dx.doi.org/10.15585/mmwr.mm7309a4

15. Estrella-Porter P, Blanco-Calvo C, Lameiras-Azevedo AS, Juaneda J, Fernandez-Martinez S, Gomez-Pajares F*, et al.* Effectiveness of nirsevimab introduction against respiratory syncytial virus in the Valencian Community: a preliminary assessment. Vaccine. 2024.03:03. doi: https://dx.doi.org/10.1016/j.vaccine.2024.05.078

16. Ezpeleta G, Navascues A, Viguria N, Herranz-Aguirre M, Juan Belloc SE, Gimeno Ballester J*, et al.* Effectiveness of nirsevimab immunoprophylaxis administered at birth to prevent infant hospitalisation for respiratory syncytial virus infection: a population-based cohort study. Vaccines (Basel). 2024.12(4):04. doi: https://dx.doi.org/10.3390/vaccines12040383

17. Fortunato F, Campanozzi A, Maffei G, Arena F, Carri VD, Rollo T*, et al.* Respiratory syncytial virus-associated hospitalizations among children: an Italian retrospective observational study. Ital J Pediatr. 2024.50(1):45. doi: https://dx.doi.org/10.1186/s13052-024-01617-w

18. Lodi L, Catamero F, Voarino M, Barbati F, Moriondo M, Nieddu F*, et al.* Epidemiology of respiratory syncytial virus in hospitalized children over a 9-year period and preventive strategy impact. Front Pharmacol. 2024.15:1381107. doi: https://dx.doi.org/10.3389/fphar.2024.1381107

19. Paireau J, Durand C, Raimbault S, Cazaubon J, Mortamet G, Viriot D*, et al.* Nirsevimab effectiveness against cases of respiratory syncytial virus bronchiolitis hospitalised in paediatric intensive care units in France, September 2023-January 2024. Influenza other respi. 2024.18(6):e13311. doi: https://dx.doi.org/10.1111/irv.13311

20. Higgins J, Thomas J, Chandler J, Cumpston M, Li T, Page M, Welch V. Cochrane Handbook for Systematic Reviews of Interventions (Version 6.4). Cochrane; 2023. Available from: https://training.cochrane.org/handbook/current.

21. NHS Centre for Reviews and Dissemination, Khan K, Ter Riet G, Glanville J, Sowden A, Kleijnen J. Undertaking systematic reviews of research on effectiveness: CRD’s guidance for those carrying out or commissioning reviews. CRD Report 4 [3rd edition]. York: 2009. Available from: http://www.york.ac.uk/inst/crd/report4.htm.

22. Centre for Reviews and Dissemination. PROSPERO: international prospective register of systematic reviews. York: CRD; 2024. [cited 10 April 2024]. Available from: http://www.crd.york.ac.uk/PROSPERO/.

23. Rethlefsen ML, Kirtley S, Waffenschmidt S, Ayala AP, Moher D, Page MJ, Koffel JB. PRISMA-S: an extension to the PRISMA Statement for Reporting Literature Searches in Systematic Reviews. Syst Rev. 2021.10(1):39. doi: 10.1186/s13643-020-01542-z

24. Page MJ, McKenzie JE, Bossuyt PM, Boutron I, Hoffmann TC, Mulrow CD*, et al.* The PRISMA 2020 statement: an updated guideline for reporting systematic reviews. BMJ. 2021.372:n71. doi: 10.1136/bmj.n71

25. Page MJ, Moher D, Bossuyt PM, Boutron I, Hoffmann TC, Mulrow CD*, et al.* PRISMA 2020 explanation and elaboration: updated guidance and exemplars for reporting systematic reviews. BMJ. 2021.372:n160. doi: 10.1136/bmj.n160

26. Clarivate. EndNote 21. Clarivate; 2023. Available from: http://endnote.com/.

27. Moola S, Munn Z, Tufanaru C, Aromataris E, Sears K, Sfetcu R*, et al.* Chapter 7: Systematic reviews of etiology and risk. In: Aromataris E, Munn Z, editors. Joanna Briggs Institute (JBI) Manual for Evidence Synthesis. JBI. 2024. Available from: https://synthesismanual.jbi.global/.

28. Higgins JP, Altman DG, Sterne JA, Cochrane Statistical Methods Group and the Cochrane Bias Methods Group. Chapter 8: Assessing risk of bias in a randomized trial. In: Higgins JP, Green S, editors. Cochrane Handbook for Systematic Reviews of Interventions version 6.4. Cochrane. 2023. Available from: https://training.cochrane.org/handbook/current/chapter-08.

# Appendix A – PRISMA Record Selection Process

Figure A.1: PRISMA flow diagram of record selection process [24]

**Identification of studies via other methods**

**Identification of studies via databases and registers**

Records removed *before screening*:

Duplicate records removed (n = )

Records removed for other reasons (n = )

Records identified from:

HTA / regulatory agency webpages (n = )

Other webpages (n = )

Reference list checking (n = )

Non-database conference searching (n = )

Other sources (n = ) [e.g. client, experts]

Records removed *before screening*:

Duplicate records removed (n = )

Records removed for other reasons (n = )

Records identified from:

Databases (n = )

Registers (n = )

**Identification**

Records excluded (n = )

Records screened (n = )

Records excluded (n = )

Records screened (n = )

**Screening**

Reports* not retrieved (n = )

Reports* sought for retrieval (n = )

Reports* not retrieved (n = )

Reports* sought for retrieval (n = )

Reports* excluded:

Reason 1 (n = )

Reason 2 (n = )

Reason 3 (n = )

etc.

Reports* excluded:

Reason 1 (n = )

Reason 2 (n = )

Reason 3 (n = )

etc.

Reports* assessed for eligibility (n = )

Reports* assessed for eligibility (n = )

**Included**

Studies included in review (n = )

Reports* of included studies (n = )

* "Note that a “report” could be a journal article, preprint, conference abstract, study register entry, clinical study report, dissertation, unpublished manuscript, government report or any other document providing relevant information": https://www.bmj.com/content/372/bmj.n71.

*Adapted from:* Page MJ, McKenzie JE, Bossuyt PM, Boutron I, Hoffmann TC, Mulrow CD, et al. The PRISMA 2020 statement: an updated guideline for reporting systematic reviews. BMJ 2021;372:n71. doi: 10.1136/bmj.n71. For more information, visit: <http://www.prisma-statement.org/>

# Appendix B – PRISMA Checklist

Table B.1: PRISMA Checklist [24]

| Section/topic | Item # | Checklist item | Location where item is reported |
| --- | --- | --- | --- |
| Title | | | |
| Title | 1 | Identify the report as a systematic review. |  |
| Abstract | | | |
| Abstract | 2 | See the PRISMA 2020 for Abstracts checklist. |  |
| Introduction | | | |
| Rationale | 3 | Describe the rationale for the review in the context of existing knowledge. |  |
| Objectives | 4 | Provide an explicit statement of the objective(s) or question(s) the review addresses. |  |
| Methods | | | |
| Eligibility criteria | 5 | Specify the inclusion and exclusion criteria for the review and how studies were grouped for the syntheses. |  |
| Information sources | 6 | Specify all databases, registers, websites, organisations, reference lists and other sources searched or consulted to identify studies. Specify the date when each source was last searched or consulted. |  |
| Search strategy | 7 | Present the full search strategies for all databases, registers and websites, including any filters and limits used. |  |
| Selection process | 8 | Specify the methods used to decide whether a study met the inclusion criteria of the review, including how many reviewers screened each record and each report retrieved, whether they worked independently, and if applicable, details of automation tools used in the process. |  |
| Data collection process | 9 | Specify the methods used to collect data from reports, including how many reviewers collected data from each report, whether they worked  independently, any processes for obtaining or confirming data from study investigators, and if applicable, details of automation tools used in  the process. |  |
| Data items | 10a | List and define all outcomes for which data were sought. Specify whether all results that were compatible with each outcome domain in each study were sought (e.g. for all measures, time points, analyses), and if not, the methods used to decide which results to collect. |  |
|  | 10b | List and define all other variables for which data were sought (e.g. participant and intervention characteristics, funding sources). Describe any assumptions made about any missing or unclear information. |  |
| Study risk of bias assessment | 11 | Specify the methods used to assess risk of bias in the included studies, including details of the tool(s) used, how many reviewers assessed each study and whether they worked independently, and if applicable, details of automation tools used in the process. |  |
| Effect measures | 12 | Specify for each outcome the effect measure(s) (e.g. risk ratio, mean difference) used in the synthesis or presentation of results. |  |
| Synthesis methods | 13a | Describe the processes used to decide which studies were eligible for each synthesis (e.g. tabulating the study intervention characteristics and comparing against the planned groups for each synthesis (item #5)). |  |
|  | 13b | Describe any methods required to prepare the data for presentation or synthesis, such as handling of missing summary statistics, or data conversions. |  |
|  | 13c | Describe any methods used to tabulate or visually display results of individual studies and syntheses. |  |
|  | 13d | Describe any methods used to synthesize results and provide a rationale for the choice(s). If meta-analysis was performed, describe the model(s), method(s) to identify the presence and extent of statistical heterogeneity, and software package(s) used. |  |
|  | 13e | Describe any methods used to explore possible causes of heterogeneity among study results (e.g. subgroup analysis, meta-regression). |  |
|  | 13f | Describe any sensitivity analyses conducted to assess robustness of the synthesized results. |  |
| Reporting bias assessment | 14 | Describe any methods used to assess risk of bias due to missing results in a synthesis (arising from reporting biases). |  |
| Certainty assessment | 15 | Describe any methods used to assess certainty (or confidence) in the body of evidence for an outcome. |  |
| Results | | | |
| Study selection | 16a | Describe the results of the search and selection process, from the number of records identified in the search to the number of studies included in the review, ideally using a flow diagram. |  |
|  | 16b | Cite studies that might appear to meet the inclusion criteria, but which were excluded, and explain why they were excluded. |  |
| Study characteristics | 17 | Cite each included study and present its characteristics. |  |
| Risk of bias in studies | 18 | Present assessments of risk of bias for each included study. |  |
| Results of individual studies | 19 | For all outcomes, present, for each study: (a) summary statistics for each group (where appropriate) and (b) an effect estimate and its precision (e.g. confidence/credible interval), ideally using structured tables or plots. |  |
| Results of syntheses | 20a | For each synthesis, briefly summarise the characteristics and risk of bias among contributing studies. |  |
|  | 20b | Present results of all statistical syntheses conducted. If meta-analysis was done, present for each the summary estimate and its precision (e.g. confidence/credible interval) and measures of statistical heterogeneity. If comparing groups, describe the direction of the effect. |  |
|  | 20c | Present results of all investigations of possible causes of heterogeneity among study results. |  |
|  | 20d | Present results of all sensitivity analyses conducted to assess the robustness of the synthesized results. |  |
| Reporting biases | 21 | Present assessments of risk of bias due to missing results (arising from reporting biases) for each synthesis assessed. |  |
| Certainty of evidence | 22 | Present assessments of certainty (or confidence) in the body of evidence for each outcome assessed. |  |
| Discussion | | | |
| Discussion | 23a | Provide a general interpretation of the results in the context of other evidence. |  |
|  | 23b | Discuss any limitations of the evidence included in the review. |  |
|  | 23c | Discuss any limitations of the review processes used. |  |
|  | 23d | Discuss implications of the results for practice, policy, and future research. |  |
| Other information | | | |
| Registration and protocol | 24a | Provide registration information for the review, including register name and registration number, or state that the review was not registered. |  |
|  | 24b | Indicate where the review protocol can be accessed, or state that a protocol was not prepared. |  |
|  | 24c | Describe and explain any amendments to information provided at registration or in the protocol. |  |
| Support | 25 | Describe sources of financial or non-financial support for the review, and the role of the funders or sponsors in the review |  |
| Competing interests | 26 | Declare any competing interests of review authors. |  |
| Availability of data, code and other materials | 27 | Report which of the following are publicly available and where they can be found: template data collection forms; data extracted from included studies; data used for all analyses; analytic code; any other materials used in the review. |  |

# Appendix C – Protocol Amendments

The protocol was agreed by email on XXXX

The following protocol amendments (PA) were made subsequent to agreement of the final protocol:

- [Summary of amendment].

| Date of amendment | Protocol Section | PA number | Description of change |
| --- | --- | --- | --- |
|  |  |  |  |
|  |  |  |  |
|  |  |  |  |
|  |  |  |  |
|  |  |  |  |
|  |  |  |  |
|  |  |  |  |
|  |  |  |  |
|  |  |  |  |
|  |  |  |  |

PA: Protocol Amendment
