# Supplementary material for: Nirsevimab for preventing respiratory syncytial virus lower respiratory tract infections in infants: a systematic review and meta-analysis
Source: Front Public Health. 2025 Oct 24;13:1641085. doi: 10.3389/fpubh.2025.1641085 (PMC12592186; doi:10.3389/fpubh.2025.1641085)
Supplement: Supplementary file 2 [file Data_Sheet_2.DOCX]

Supplementary Material 2: Literature Searches

# Resources Searched

We conducted the literature search in the databases and information resources shown in Table 1.

**Table 1: Databases and information sources searched**

| **Resource** | **Interface / URL** |
| --- | --- |
| **Databases** | |
| MEDLINE(R) ALL | OvidSP |
| Embase | OvidSP |
| Cochrane Database of Systematic Reviews (CDSR) | Cochrane Library/Wiley |
| Cochrane Central Register of Controlled Trials (CENTRAL) | Cochrane Library/Wiley |
| HTA Database | https://database.inahta.org/ |
| Conference Proceedings Citation Index – Science (CPCI-S) | Web of Science |
|  |  |
| **Trials Registers** | |
| ClinicalTrials.gov | https://clinicaltrials.gov/ |
| WHO International Clinical Trials Registry Platform (ICTRP) | https://trialsearch.who.int/ |
|  |  |
| **HTA / regulatory agency webpages** | |
| Drugs@FDA (Food and Drug Administration) | https://www.accessdata.fda.gov/scripts/cder/daf/index.cfm |
| Centers for Disease Control and Prevention (CDC) Advisory Committee on Immunization Practices (ACIP) | https://stacks.cdc.gov/advancesearch |
| European Medicines Agency (EMA) medicines webpages | https://www.ema.europa.eu/en |
| National Institute for Health and Care Excellence (NICE) webpages | https://www.nice.org.uk/ |
| Canadian Agency for Drugs and Technologies in Health (CADTH) webpages (since renamed Canada's Drug Agency CDA-AMC) | https://www.cadth.ca/ |
| Institute for Clinical and Economic Review (ICER) webpages | https://icer-review.org/ |
|  |  |
| **Non-database conference searches** | See details in search methods |
| **Reference list checking** | n/a |

The trials register sources listed above (ClinicalTrials.gov and ICTRP) were searched to identify information on studies in progress. A number of data providers provide data to WHO for inclusion in ICTRP, including the EU Clinical Trials Register (EU-CTR).

In addition to searching the HTA database, targeted searches of the listed technology assessment and regulatory agency websites were conducted:

- Drugs@FDA for medical, statistical and other reviews.
- Centers for Disease Control and Prevention (CDC) Advisory Committee on Immunization Practices (ACIP) for scientific data presented at ACIP meetings (2023 onwards)
- European Medicines Agency (EMA) medicines webpages for European public assessment reports (EPARs).
- National Institute for Health and Care Excellence (NICE) webpages for company submissions to NICE, Final Appraisal Determination documents, Evidence Review Group (ERG) reports (for single technology assessments only), and assessment reports.
- Canadian Agency for Drugs and Technologies in Health (CADTH) webpages (since renamed Canada's Drug Agency CDA-AMC) for CADTH Clinical Guidance, and CADTH Final Recommendations associated with Reimbursement Reviews.
- Institute for Clinical and Economic Review (ICER) webpages for Final Evidence Reports (or Draft Evidence Reports if final was not yet available) and Evidence Presentation.

Reflecting the eligibility criteria, CPCI-S search results and records indexed in Embase as conference abstracts were restricted to studies published from 2021 to date. Reflecting the eligibility criteria, records indexed as preprints were excluded from the Embase search results.

Recent research published as conference abstracts was identified by searching Embase (which indexes a significant number of conference publications) and CPCI-S (which is a conference proceedings citation index for science disciplines). The following conferences were identified as highly relevant:

- ISPOR Europe
- ISPOR US
- European Society for Paediatric Infectious Diseases (ESPID) Annual Meeting
- ReSViNET Conference

We ascertained if records for these conferences (held in the between 2021 and the search date) were included in Embase or CPCI-S. Records were not found in Embase or CPCI-S for the conferences listed below. For these conferences we sought conference proceedings via the conference organiser.

- ISPOR Europe 2024
- European Society for Paediatric Infectious Diseases (ESPID) Annual Meeting
- ReSViNET Conference

ISPOR Europe 2024 abstracts were retrieved via the ISPOR presentations database on the ISPOR website. The ReSViNET Conference was not held in 2022. Abstract books for the other ReSViNET conferences and the ESPID Annual Meeting were downloaded and searched for relevant abstracts. For further details of these searches, please see below.

We also checked the included studies list of any retrieved relevant systematic reviews published in the last three years for any eligible studies that may have been missed by the database searches.

For each paper that was selected for inclusion in the review, a check was made to establish if any of the following notices were associated with the included paper: retraction notice, erratum notice, corrected and republished paper notice, expression of concern notice. The check was conducted via the PubMed record for the paper or (if no PubMed record was found) via the journal webpage for the paper. If a relevant notice was associated with an included paper, the notice was assessed by the review team.

**Search strategies**

1. **Source:** **MEDLINE ALL**

Interface / URL: OvidSP

Database coverage dates: 1946 to 25 November 2024

Search date: 26 November 2024

Retrieved records: 223

Search strategy:

1 nirsevimab*.ti,ab,kf,rn,nm,ot. 217

2 (beyfortus*2 or nirsevumab or MEDI8897*2 or medi 8897*2 or 1989556-22-0 or VRN8S9CW5V or MED 18897*2 or MED18897*2 or sp 0232*2 or sp 232*2 or sp0232*2 or sp232*2).ti,ab,kf,rn,nm,ot. 90

3 or/1-2 236

4 exp animals/ not humans/ 5280703

5 (news or editorial).pt. 942129

6 or/4-5 6195066

7 3 not 6 223

1. **Source: Embase**

Interface / URL: OvidSP

Database coverage dates: 1974 to 25 November 2024

Search date: 26 November 2024

Retrieved records: 416

Search strategy:

1 nirsevimab/ 409

2 nirsevimab*.ti,ab,kf,dq,rn,tn,dy,ot. 433

3 (beyfortus*2 or nirsevumab or MEDI8897*2 or medi 8897*2 or 1989556-22-0 or VRN8S9CW5V or MED 18897*2 or MED18897*2 or sp 0232*2 or sp 232*2 or sp0232*2 or sp232*2).ti,ab,kf,dq,rn,tn,dy,ot. 422

4 or/1-3 461

5 (animal/ or animal experiment/ or animal model/ or animal tissue/ or nonhuman/) not exp human/ 7133310

6 editorial.pt. 829708

7 preprint.pt. 148193

8 or/5-7 8042851

9 4 not 8 426

10 conference abstract.pt. 5286236

11 9 not 10 386

12 limit 10 to yr="2021 -current" 1108712

13 9 and 12 30

14 11 or 13 416

1. **Source:** **Cochrane Database of Systematic Reviews (CDSR)**

Interface / URL: Cochrane Library / Wiley

Database coverage dates: Not found. Issue searched: Issue 11 of 12, November 2024

Search date: 26 November 2024

Retrieved records: 0

Search strategy:

#1 nirsevimab*:ti,ab,kw 32

#2 (beyfortus* or nirsevumab or MEDI8897* or "medi 8897" or "medi 8897r" or "medi 8897tm" or "1989556 22 0" or VRN8S9CW5V or "MED 18897" or "MED 18897r" or "MED 18897tm" or MED18897* or "sp 0232" or "sp 0232r" or "sp 0232tm" or "sp 232" or "sp 232r" or "sp 232tm" or sp0232* or sp232*):ti,ab,kw 18

#3 #1 or #2 in Cochrane Reviews, Cochrane Protocols 0

1. **Source:** **Cochrane Central Register of Controlled Trials (CENTRAL)**

Interface / URL: Cochrane Library / Wiley

Database coverage dates: Not found. Issue searched: Issue 10 of 12, October 2024

Search date: 26 November 2024

Retrieved records: 44

Search strategy:

#1 nirsevimab* 34

#2 (beyfortus* or nirsevumab or MEDI8897* or "medi 8897" or "medi 8897r" or "medi 8897tm" or "1989556 22 0" or VRN8S9CW5V or "MED 18897" or "MED 18897r" or "MED 18897tm" or MED18897* or "sp 0232" or "sp 0232r" or "sp 0232tm" or "sp 232" or "sp 232r" or "sp 232tm" or sp0232* or sp232*) 19

#3 #1 or #2 in Trials 44

1. **Source: HTA database**

Interface / URL: https://database.inahta.org/

Database coverage dates: Information not found. The former database was produced by the CRD until March 2018, at which time the addition of records was stopped as INAHTA was in the process of rebuilding the new database platform. In July 2019, the database records were exported from the CRD platform and imported into the new platform that was developed by INAHTA. The rebuild of the new platform was launched in June 2020.

Search date: 26 November 2024

Retrieved records: 14

Search strategy:

The search interface for this database cannot interpret terms of only two characters in length. Some of the terms in the translation were therefore amended to account for this. For example "sp 232" was translated to "232".

#1 nirsevimab* 2

#2 (beyfortus* OR nirsevumab OR MEDI8897* OR "medi 8897" OR "medi 8897r" OR "medi 8897tm" OR "1989556" OR VRN8S9CW5V OR "MED 18897" OR "MED 18897r" OR "MED 18897tm" OR MED18897* OR "0232" OR "0232r" OR "0232tm" OR "232" OR "232r" OR "232tm" OR sp0232* OR sp232*) 12

#3 #1 OR #2 14

1. **Source: Conference Proceedings Citations Index – Science (CPCI-S)**

Interface / URL:

Database coverage dates: 1990 to present

Search date: 26 November 2024

Retrieved records: 1

Search strategy:

The "Exact search" option was used for all search lines. A publication date limit was applied to line 3 as follows: 2021-01-01 to 2024-11-26

1 TS=nirsevimab* 1

2 TS=(beyfortus* OR nirsevumab OR MEDI8897* OR "medi 8897*" OR 1989556-22-0 OR VRN8S9CW5V OR "MED 18897*" OR MED18897* OR "sp 0232*" OR "sp 232*" OR sp0232* OR sp232*) 0

3 #1 OR #2 1

1. **Source:** **ClinicalTrials.gov**

Interface / URL: https://www.clinicaltrials.gov/

Database coverage dates: Information not found. ClinicalTrials.gov was created as a result of the Food and Drug Administration Modernization Act of 1997 (FDAMA). The site was made available to the public in February 2000.

Search date: 26 November 2024

Retrieved records: 21

Search strategy:

The following search was conducted at the URL above. The search was run in the "other terms" field.

(nirsevimab OR nirsevimabe OR beyfortus OR beyfortusr OR beyfortustm OR nirsevumab OR MEDI8897 OR MEDI8897r OR MEDI8897tm OR "medi 8897" OR "medi 8897r" OR "medi 8897tm" OR "1989556 22 0" OR VRN8S9CW5V OR "MED 18897" OR "MED 18897r" OR "MED 18897tm" OR MED18897 OR MED18897r OR MED18897tm OR "sp 0232" OR "sp 0232r" OR "sp 0232tm" OR "sp 232" OR "sp 232r" OR "sp 232tm" OR sp0232 OR sp0232r OR sp0232tm OR sp232 OR sp232r OR sp232tm)

1. **Source:** **WHO International Clinical Trials Registry Portal (ICTRP)**

Interface / URL: https://trialsearch.who.int/

Database coverage dates: Information not found. On the date of search, files had been imported from data providers between 14 October 2024 and 18 November 2024.

Search date: 26 November 2024

Retrieved records: 30

Search strategy:

The following search was conducted using the search interface at the above URL. 'Without Synonyms' was selected.

(nirsevimab* OR beyfortus* OR nirsevumab OR MEDI8897* OR "medi 8897*" OR "1989556 22 0" OR VRN8S9CW5V OR "MED 18897*" OR MED18897* OR "sp 0232*" OR "sp 232*" OR sp0232* OR sp232*)

= 74 records for 30 trials

1. **Source: Drugs@FDA**

Interface / URL: https://www.accessdata.fda.gov/scripts/cder/daf/index.cfm

Database coverage dates: 1938 to present

Search date: 26 November 2024

Retrieved records: 5

Search strategy:

The following documents were sought for the drug of interest: Medical Reviews, Clinical Reviews, Statistical Reviews, Other Reviews.

The search interface at the following URL was used: <https://www.accessdata.fda.gov/scripts/cder/daf/index.cfm>

Separate searches were conducted on each drug name shown below.

For each search:

- Each returned drug name was selected to expand the result
- Each of the drugs shown on expansion was selected to display details
- The link to 'Reviews' was selected to view documents

PDFs for Medical reviews, Clinical Reviews, Statistical Reviews or Other Reviews on the correct indication were retrieved for further assessment. 5 documents were retrieved. Duplicate documents were not retrieved.

nirsevimab

nirsevimabe

beyfortus

nirsevumab

MEDI8897

medi 8897

1989556-22-0

VRN8S9CW5V

MED 18897

MED18897

sp 0232

sp 232

sp0232

sp232

1. **Source: Centers for Disease Control and Prevention (CDC) Advisory Committee on Immunization Practices (ACIP)**

Interface / URL: https://stacks.cdc.gov/advancesearch

Database coverage dates: Information not found

Search date: 26 November 2024

Retrieved records: 27

Search strategy:

Scientific data presented at ACIP meeting between 2023 and the search date was sought by entering the following search terms at the URL above. The searches were limited to Advisory Committee on Immunization Practices (ACIP) using the built in "Collections" filter.

27 results from 2023 and 2024 were downloaded for further assessment.

nirsevimab

nirsevimabe

beyfortus

nirsevumab

MEDI8897

medi 8897

1989556-22-0

VRN8S9CW5V

MED 18897

MED18897

sp 0232

sp 232

sp0232

sp232

1. **Source:** **European Medicines Agency (EMA) medicines webpages**

Interface / URL: https://www.ema.europa.eu/en

Database coverage dates: n/a

Search date: 26 November 2024

Retrieved records: 5

Search strategy:

The following documents were sought for the drug of interest: 'EPAR - Public Assessment Report'. 'Assessment Report' associated documents with variant titles were also retrieved (e.g. 'EPAR - Assessment Report', 'EPAR - Assessment Report – Variation', 'EPAR procedural steps taken', EPAR – Scientific Conclusions', 'EPAR – Refusal Assessment Report' and 'EPAR - Assessment Report – Article [#]', 'EPAR - Assessment Report – Extension' etc.)

Documents found under both 'Initial marketing-authorisation documents' and ' Changes since initial authorisation of medicine' were retrieved.

The search interface at the following URL was used: <https://www.ema.europa.eu/en/medicines>. Separate searches were conducted on each drug name shown below. Duplicate documents were not retrieved.

5 documents were retrieved

nirsevimab

nirsevimabe

beyfortus

nirsevumab

MEDI8897

medi 8897

1989556-22-0

VRN8S9CW5V

MED 18897

MED18897

sp 0232

sp 232

sp0232

sp232

1. **Source:** **Canadian Agency for Drugs and Technologies in Health (CADTH) webpages**

Interface / URL: https://www.cadth.ca/

Database coverage dates: n/a

Search date: 26 November 2024

Retrieved records: 2

Search strategy:

The following documents were sought for the drug of interest: CADTH Clinical Guidance, and CADTH Final Recommendations associated with Reimbursement Reviews.

The site-wide search interface was used at: <https://www.cadth.ca/>. Separate searches were conducted on each drug name shown below. Two documents were downloaded for further assessment. Duplicate documents were not retrieved.

Since running the searches CADTH has become Canada's Drug Agency (CDA-AMC) and the URL is now: <https://www.cda-amc.ca/>

nirsevimab

nirsevimabe

beyfortus

nirsevumab

MEDI8897

medi 8897

1989556-22-0

VRN8S9CW5V

MED 18897

MED18897

sp 0232

sp 232

sp0232

sp232

1. **Source:** **National Institute for Health and Care Excellence (NICE) webpages**

Interface / URL: https://www.nice.org.uk/

Database coverage dates: n/a

Search date: 26 November 2024

Retrieved records: 0

Search strategy:

The following documents were sought for the drug of interest: company submissions to NICE, Final Appraisal Determination documents, Evidence Review Group (ERG) reports (for single technology assessments only), and assessment reports (for multiple technology appraisals only).

The site-wide search interface was used at: <https://www.nice.org.uk/>. Separate searches were conducted on each term shown below. No results were retrieved.

nirsevimab

nirsevimabe

beyfortus

nirsevumab

MEDI8897

medi 8897

1989556-22-0

VRN8S9CW5V

MED 18897

MED18897

sp 0232

sp 232

sp0232

sp232

1. **Source:** **Institute for Clinical and Economic Review webpages**

Interface / URL: https://icer-review.org/

Database coverage dates: n/a

Search date: 26 November 2024

Retrieved records: 0

Search strategy:

The following documents were sought for the drug of interest: for Final Evidence Reports (or Draft Evidence Reports if final is not yet available) and Evidence Presentation (if available).

Each of the following terms was searched on the website URL noted above.

nirsevimab

nirsevimabe

beyfortus

nirsevumab

MEDI8897

medi 8897

1989556

VRN8S9CW5V

MED 18897

MED18897

sp 0232

sp 232

sp0232

sp232

The filter 'Diseases & Conditions' was used to identify assessments in RSV.

No results were retrieved.

1. **Source:** **ISPOR Europe conferences 2021 to 2024**

Embase and CPCI-S were checked to ascertain if they included records for these conferences for the period required (conferences held between 2021 and 26 November 2024). Records for the conferences held in 2021, 2022 and 2023 were found in Embase. Our assumption was that the relevant conferences were fully indexed in the database at the time of the search, and no additional searches were required.

For the ISPOR EU 2024 conference we conducted searches at the following URL: <https://www.ispor.org/heor-resources/presentations-database/search>

The following syntax was used in the "keyword" search:

(nirsevimab* OR beyfortus* OR nirsevumab OR MEDI8897* OR "medi 8897*" OR "1989556 22 0" OR VRN8S9CW5V OR "MED 18897*" OR MED18897* OR "sp 0232*" OR "sp 232*" OR sp0232* OR sp232*)

Results were limited to the 2024 conference. Nine records were retrieved and downloaded for further assessment.

1. **Source:** **ISPOR US conferences 2021 to 2024**

Embase and CPCI-S were checked to ascertain if they included records for these conferences for the period required (conferences held between 2021 and 26 November 2024). Records for the conference of interest were found in Embase. Our assumption was that the relevant conferences were fully indexed in the database at the time of the search, and no additional searches were required.

1. **Source:** **European Society for Paediatric Infectious Diseases (ESPID) Annual Meeting**

Search date: 26 November 2024

Retrieved records: 37

Search strategy:

Meeting abstract books for the years 2021 to 2024 inclusive were downloaded from the following site: <https://espidmeeting.org/past-abstracts/>

The PDFs were searched using CTRL+F for the terms below. Abstracts including these terms were retained for further assessment. Duplicate abstracts were not retained.

nirsevimab

nirsevimabe

beyfortus

nirsevumab

MEDI8897

medi 8897

1989556

VRN8S9CW5V

MED 18897

MED18897

sp 0232

sp 232

sp0232

sp232

Abstracts for further assessment:

2021 = 0

2022 = 3

2023 = 8

2024 = 26

Total = 37

1. **Source:** **ReSViNET Conference (RSVVW)**

Search date: 26 November 2024

Retrieved records: 31

Search strategy:

Meeting abstract books for the years 2021, 2023 and 2024 were downloaded from the following site: <https://resvinet.org/conferences/>. No conference took place in 2022

The PDFs were searched using CTRL+F for the terms below. Abstracts including these terms were retained for further assessment. Duplicate abstracts were not retained.

nirsevimab

nirsevimabe

beyfortus

nirsevumab

MEDI8897

medi 8897

1989556

VRN8S9CW5V

MED 18897

MED18897

sp 0232

sp 232

sp0232

sp232

Abstracts for further assessment:

2021 = 3

2023 = 11

2024 = 17

Total = 31

## Supplementary Figures

**
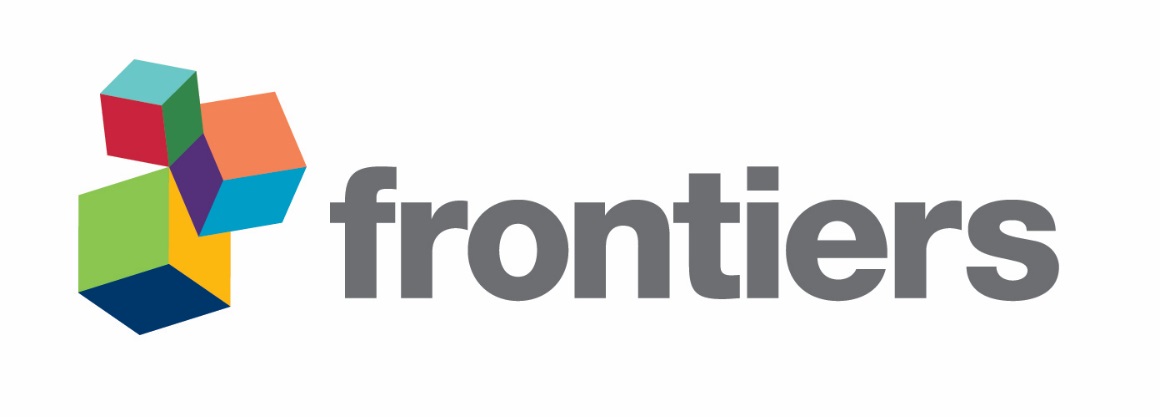
**

**Supplementary Figure 1.** The figure legends are required to have the same font as the main text, 12 point normal Times New Roman, single spaced. Please use a single paragraph for each legend and prepare the figures keeping in mind the PDF layout.
