# Supplementary material for: Nirsevimab for preventing respiratory syncytial virus lower respiratory tract infections in infants: a systematic review and meta-analysis
Source: Front Public Health. 2025 Oct 24;13:1641085. doi: 10.3389/fpubh.2025.1641085 (PMC12592186; doi:10.3389/fpubh.2025.1641085)
Supplement: Supplementary file 3 [file Data_Sheet_3.DOCX]

Supplementary Material 3: Outcome Data

**Table 1: Secondary care RSV outcomes**

| Study | Design | Group | Follow-up | Events/ Sample size (incidence) | Effect measure* | Effectiveness (%) |
| --- | --- | --- | --- | --- | --- | --- |
|  |  |  |  |  | Estimate (95% CI) | Estimate (95% CI) |
| **RSV ARI-related hospitalisation** | | | | | | |
| Moline 2024 | Case-control study | Cases | NA | 6/407 (NA)^4^ | aOR Estimate NR | 90 (75 to 96)^1^ |
|  |  | Controls |  | 53/292 (NA)^4^ |  |  |
| Lefferts 2024 | Case-control study | Cases | NA | 3/20 (NA)^4^ | aOR Estimate NR | 89 (32 to 98)^2^ |
|  |  | Controls |  | 19/29 (NA)^4^ |  |  |
| **RSV LRTI-related hospitalisation** | | | | | | |
| Ares-Gómez 2024 | Cohort study | Nirsevimab | Median 81 days (IQR 68-87) | 30/9,408 (0.3%) | aIRR 0.18 (0.10 to 0.34)^3^  aHR 0.18 (0.09 to 0.33)^4^ | 82.0 (65.6 to 90.2)^3^  82.4 (66.9 to 90.6)^4^ |
|  |  | No nirsevimab |  | 16/851 (1.9%) |  |  |
| Ezpeleta 2024 | Cohort study | Nirsevimab | Up to 4 months | 8/1,083 (0.74%) | aHR 0.113 (0.042 to 0.304)^5^ | 88.7 (69.6 to 95.8)^5^ |
|  |  | No nirsevimab |  | 8/94 (8.51%) |  |  |
| Lopez-Lacort 2024 | Case-control study | Cases | Up to 3 months | 56/95 (NA)^4^ | aOR | 70.2 (38.3 to 88.5)^6^ |
|  |  | Controls |  | 59/71 (NA)^4^ |  |  |
| Hsiao 2024 | Cohort study | Nirsevimab | NR | NR/15647 | OR 0.11 (0.01 to 0.85) | 89.0 (15.0 to 99.0)^11^ |
|  |  | No nirsevimab |  | NR/16253 |  |  |
| Del Buey 2024 | Cohort study | Nirsevimab | NR | Month 1: 34/NR (NR)  Month 2: 50/NR (NR)  Month 3: 33/NR (NR)  Month 4: 8/NR (NR)  Month 5: 7/NR (NR) | NR | Month 1: 93.6 (89.7 to 96.1)  Month 2: 92.5 (89.9 to 94.4)  Month 3: 91.1 (86.9 to 94.0)  Month 4: 89.5 (79.8 to 94.6)  Month 5: 87.6 (67.7 to 95.3) |
|  |  | No nirsevimab |  | Month 1: 46/NR (NR)  Month 2: 157/NR (NR)  Month 3: 138/NR (NR)  Month 4: 24/NR (NR)  Month 5: 8/NR(NR) |  |  |
| **RSV LRTI-related emergency room (ER) visits** | | | | | | |
| Ezpeleta 2024 | Cohort study | Nirsevimab | Up to 4 months | 11/1,083 (1.02%) | aHR 0.121 (0.049 to 0.297)^6^ | 87.9 (70.3 to 95.1)^6^ |
|  |  | No nirsevimab |  | 9/94 (9.57%) |  |  |
| Molina Gutierrez 2024 | Cross-sectional study | Post-nirsevimab | Up to 12 weeks | 52/ NR | NR | NR |
|  |  | Pre-nirsevimab |  | 212/ NR |  |  |
| **RSV LRTI-related intensive care unit (ICU) admission** | | | | | | |
| Ares-Gómez 2024 | Cohort study | Nirsevimab | Median 81 days (IQR 68-87) | 10/9,408 (0.1%) | NR^7^ | NR^7^ |
|  |  | No nirsevimab |  | 0/ 851 (NA) |  |  |
| Ezpeleta 2024 | Cohort study | Nirsevimab | Up to 4 months | 3/1,083 (0.28%) | aHR 0.141 (0.023 to 0.868)^6^ | 85.9 (13.2 to 97.7)^6^ |
|  |  | No nirsevimab |  | 2/94 (2.13%) |  |  |
| **RSV LRTI hospitalisation with oxygen support** | | | | | | |
| Ares-Gómez 2024 | Cohort study | Nirsevimab | Median 81 days (IQR 68-87) | 15/9,408 (0.2%) | aIRR 0.13 (0.06 to 0.31)^3^  aHR 0.13 (0.06 to 0.30)^4^ | 86.9 (69.1 to 94.2)^3^  87.1 (70.2, 94.4)^4^ |
|  |  | No nirsevimab |  | 10/851 (1.2%) |  |  |
| **RSV LRTI hospitalisation with invasive mechanical ventilation** | | | | | | |
| Ares-Gómez 2024 | Cohort study | Nirsevimab | Median 81 days (IQR 68-87) | 0/9408 (NA) | NR^7^ | NR^7^ |
|  |  | No nirsevimab |  | 0/851 (NA) |  |  |
| **RSV LRTI hospitalisation with non-invasive mechanical ventilation** | | | | | | |
| Ares-Gómez 2024 | Cohort study | Nirsevimab | Median 81 days (IQR 68-87) | 7/9,408 (0.1%) | NR^7^ | NR^7^ |
|  |  | No nirsevimab |  | 0/851 (NA) |  |  |
| **Hospitalisation for RSV-bronchiolitis** | | | | | | |
| Assad 2024 | Case-control study | Case | NA | 60/690 (NA)^4^ | aOR NR | 83.0 (73.4 to 89.2)^8^ |
|  |  | Control |  | 97/345 (NA)^4^ |  |  |
| Coma 2024 | Cohort study | Nirsevimab | Up to 4 months | 52/23,127 (0.22%) | aHR 0.124 (0.086 to 0.179)^9^ | 87.6 (82.1 to 91.4)^9^ |
|  |  | No nirsevimab |  | 76/3,3,398 (2.24%) |  |  |
| Consolati 2024 | Cohort study | Nirsevimab | Up to 8 weeks | 0/369 (NA) | NR^7^ | NR^7^ |
|  |  | No nirsevimab |  | 14/168 (8.3) |  |  |
| Aguera 2024 | Case-control study | Case | NA | NR/141 | aOR NR | 81.0 (60.9 to 90.7)^10^ |
|  |  | Control |  | NR/93 |  |  |
| Carbajal 2024 | Case-control study | Case | NA | NR | aOR NR | 83 (72 to 90)^13^ |
|  |  | Control |  | NR |  |  |
| **ICU admission for RSV-bronchiolitis** | | | | | | |
| Assad 2024 | Case-control study | Case | NA | 27/193 (NA)^4^ | aOR NR | 69.6 (42.9 - 83.8)^8^ |
|  |  | Control |  | 47/146 (NA)^4^ |  |  |
| Coma 2024 | Cohort study | Nirsevimab | Up to 4 months | 8/23,127 (0.03%) | aHR 0.099 (0.041 to 0.237)^9^ | 90.1 (76.3 to 95.9)^9^ |
|  |  | No nirsevimab |  | 17/3,398 (0.05%) |  |  |
| Paireau 2024 | Case-control study | Case | NA | 37/238 (NA)^4^ | aOR NR | 75.9 (48.5 to 88.7)^12^ |
|  |  | Control |  | 21/50 (NA)^4^ |  |  |
| Carbajal 2024 | Case-control study | Case | NA | NR | aOR NR | 67 (–100 to 95)^13^ |
|  |  | Control |  | NR |  |  |

*Effect measure from which effectiveness estimate was derived

aIRR – adjusted incidence rate ratio; aHR – adjusted hazard ratio; aOR – adjusted odds ratio.

^1^Regression models controlled for age at enrollment in months, month of illness, enrollment site, and presence of one or more high-risk medical conditions for severe RSV disease

^2^Regression models were adjusted for age in months at medical visit, sex, calendar month, residence community type, and presence of one or more high-risk underlying condition

^3^Poisson regression models adjusted for enrolment group (catch-up and seasonal), sex, and health district area

^4^Cox proportional hazards models adjusted for enrolment group (catch-up and seasonal), sex, and health district area

^5^Adjusted for sex and week of birth

^6^Bayesian logistic regression considering hospital as a random effect

^7^Due to 0 events in the nirsevimab and/or control group

^8^The final multivariate regression model was adjusted for sex, gestational age at birth (as a continuous variable), birth weight, and risk factors for severe bronchiolitis

^9^Cox multivariate regression models were adjusted for any confounders with a standardized mean difference (SMD) between nirsevimab and control groups of >0.1

^10^Adjusted for age, weight, and the presence of at least one preexisting condition as fixed effects; the month of admission and hospital were treated as random effects

^11^Calculated from aOR

^12^Logistic regression model adjusted for age group (0–3 months, > 3 months), sex, presence of comorbidities, prematurity and time period

^13^Adjusted for the week of the paediatric emergency department visit.

**Table 2: Primary care RSV outcomes**

| Study | Design | Group | Follow-up | Events/ Sample size (incidence) | Effect measure* | Effectiveness |
| --- | --- | --- | --- | --- | --- | --- |
|  |  |  |  |  | Estimate (95% CI) | Estimate (95% CI) |
| **RSV ARIs in public health centres** | | | | | | |
| Estrella-Porter 2024 | Cohort study | Nirsevimab | Up to 14 weeks | 168/24,223 (0.69%) | aOR 0.26 (0.20 to 0.35) | 74 (65 to 80)^1^ |
|  |  | No nirsevimab |  | 72/3,139 (2.29%) |  |  |
| **RSV-ARI primary care attendance** | | | | | | |
| Coma 2024 | Cohort study | Nirsevimab | Up to 4 months | 71/23,127 (0.31%) | aHR 0.311 (0.200 to 0.483) | 68.9 (51.7 to 80)^2^ |
|  |  | No nirsevimab |  | 31/3,398 (0.91%) |  |  |
| **RSV-LRTI primary care attendance** | | | | | | |
| Lopez-Lacort 2024b | Case control study | Case | NA | 33/44 (NA)^3^ | aOR (NR) | 75.8 (40.4 to 92.7) |
|  |  | Control |  | 108/116 (NA)^3^ |  |  |
| **RSV-bronchiolitis primary care attendance** | | | | | | |
| Del Buey 2024 | Cohort study | Nirsevimab | NR | Month 1: 199/NR (NR)  Month 2: 589/NR (NR)  Month 3: 605/NR (NR)  Month 4: 559/NR (NR)  Month 5: 735/NR (NR) | NR | Month 1: 69.0 (63.5, 73.7)  Month 2: 60.9 (55.0, 65.9) Month 3: 50.6 (43.6, 56.7) Month 4: 37.5 (27.6, 46.1)  Month 5: 21.1 (5.5, 34.1) |
|  |  | No nirsevimab |  | Month 1: 285/NR (NR)  Month 2: 490/NR (NR)  Month 3: 430/NR (NR)  Month 4: 200/NR (NR)  Month 5: 171/NR (NR) |  |  |
| Lassaued 2024 | Case control study | Case | NA | 62/453 (NA) ^3^ | NR | 79.7 (67.7 to 87.3)^4^ |
|  |  | Control |  | 177/430 (NA)^3^ |  |  |

*Effect measure from which effectiveness estimate was derived

aIRR=adjusted incidence rate ratio; aHR=adjusted hazard ratio

^1^Poisson regression models adjusted for enrolment group (catch-up and seasonal), sex, and health district area

^1^Adjusted for controlling for breastfeeding intention, country of origin of the mother, gestational weeks, and campaign group

^2^Cox multivariate regression models were adjusted for any confounders with a standardized mean difference (SMD) between nirsevimab and control groups of >0.1

^3^Events are the number of infants who had been given nirsevimab. The sample size is the number or infants in the case or control group. Incidence cannot be calculated for case-control studies.

^4^Multivariate regression model was adjusted for age, sex, birth term, birth weight, previous bronchiolitis, number of children per household, month of diagnosis, childcare settings, and region

**Table 3: Medically attended/ emergency department RSV outcomes**

| Study | Design | Group | Follow-up | Events/ Sample size (incidence) | Effect measure* | Effectiveness |
| --- | --- | --- | --- | --- | --- | --- |
|  |  |  |  |  | Estimate (95% CI) | Estimate (95% CI) |
| **Medically attended RSV-ARIs** | | | | | | |
| Lefferts 2024^1^ | Case control study | Case | NA | 8/39 (NA)^2^ | NR | 76 (42–90)^3^ |
|  |  | Control |  | 153/253 (NA)^2^ |  |  |
| **Medically attended RSV-LRTIs** | | | | | | |
| Hsiao 2024^4^ | Cohort study | Nirsevimab | NR | 35/1,5647 (0.22%) | NR | 87.2 (81.7 to 91.1) |
|  |  | No nirsevimab |  | 462/1,6253 (2.84%) |  |  |
| **RSV LRTI-related ED attendance** | | | | | | |
| Ezpeleta 2024 | Cohort study | Nirsevimab | Up to 4 months | 11/1,083 (1.02%) | aHR 0.121 (0.049 to 0.297)^5^ | 87.9 (70.3 to 95.1)^5^ |
|  |  | No nirsevimab |  | 9/94 (9.57%) |  |  |
| **RSV-bronchiolitis ED attendance** | | | | | | |
| Carbajal 2024 | Case control study | Case | NA | NR | NR | 83 (CI 71−90)^6^ |
|  |  | Control |  | NR |  |  |
| Del Buey 2024 | Cohort study | Nirsevimab | NR | Month 1: 118/NR (NR)  Month 2: 293/NR (NR)  Month 3: 321/NR (NR)  Month 4: 177/NR (NR)  Month 5: 187/NR (NR) | NR | Month 1: 66.7 (61.0, 71.6)  Month 2: 58.1 (53.5, 62.3)  Month 3: 47.3 (41.2, 52.9)  Month 4: 33.8 (21.8, 43.9)  Month 5: 16.7 (−5.9, 34.5) |
|  |  | No nirsevimab |  | Month 1: 139/NR (NR)  Month 2: 325/NR (NR)  Month 3: 327/NR (NR)  Month 4: 73/NR (NR)  Month 5: 44/NR (NR) |  |  |

*Effect measure from which effectiveness estimate was derived

aIRR=adjusted incidence rate ratio; aHR=adjusted hazard ratio

^1^Hospitalisation, emergency department consultation or outpatient clinic visits.

^2^Events are the number of infants who had been given nirsevimab. The sample size is the number or infants in the case or control group. Incidence cannot be calculated for case-control studies.

^3^Regression models were adjusted for age in months at medical visit, sex, calendar month, residence community type, and presence of one or more high-risk underlying condition

^4^Medical encounters for RSV-LRTIs in any setting.

^5^Adjusted for sex and week of birth

^6^adjusted for the week of the paediatric emergency department visit

**Table 4: Non-RSV specific outcomes**

| Study | Design | Group | Follow-up | Events/ Sample size (incidence) | Effect measure* | Effectiveness |
| --- | --- | --- | --- | --- | --- | --- |
|  |  |  |  |  | Estimate (95% CI) | Estimate (95% CI) |
| **All cause hospitalisation** | | | | | | |
| Ares-Gómez 2024 | Cohort study | Nirsevimab | Median 81 days (IQR 68-87) | 289/ 9,072 (3.2%) | aIRR 0.34 (0.26 to 0.44)^1^  aHR 0.32 (0.25 to 0.42)^2^ | 66.2 (56.0 to 73.7)^1^  67.7 (58.2 to 75.1)^2^ |
|  |  | No nirsevimab |  | 77/ 817 (9.4%) |  |  |
| **ARI-related hospitalisation** | | | | | | |
| Estrella-Porter 2024 | Cohort study | Nirsevimab | Up to 14 weeks | 218/24,223 (0.9%) | OR 0.57 (0.54 to 0.6) | 42.7 (39.8 to 45.5) |
|  |  | No nirsevimab |  | 49/3,139 (1.6%) |  |  |
| **LRTI-related hospitalisation** | | | | | | |
| Ares-Gómez 2024 | Cohort study | Nirsevimab | Median 81 days (IQR 68-87) | 150/9,237 (1.6%) | aIRR 0.31 (0.22 to 0.44)^1^  aHR 0.31 (0.22 to 0.44)^2^ | 69.2 (55.9 to 78.0)  69.3 (56.4 to 78.4) |
|  |  | No nirsevimab |  | 43/826 (5.2%) |  |  |
| **Bronchiolitis-related hospitalisation** | | | | | | |
| Carbajal 2024 | Case-control study | Case | NA | NR | NR | 59 (42 to 71) |
|  |  | Control |  | NR |  |  |
| **Bronchiolitis-related primary care presentation** | | | | | | |
| Coma 2024 | Cohort study | Nirsevimab | Up to 4 months | 1,560/23,127 (6.75%) | aHR 0.519 (0.467 to 0.576)^3^ | 48.1 (42.4 to 53.3)^3^ |
|  |  | No nirsevimab |  | 617/3,398 (18.16%) |  |  |
| **Bronchiolitis-related ED visits** | | | | | | |
| Coma 2024 | Cohort study | Nirsevimab | Up to 4 months | 604/ 23,127 (2.61%) | aHR 0.446 (0.385 to 0.516)^3^ | 55.4 (48.4 to 61.5)^3^ |
|  |  | No nirsevimab |  | 354/3,398 (10.42%) |  |  |
| Carbajal 2024 | Case control study | Case | NA | NR | NR | 47 (33 to 58)^4^ |
|  |  | Control |  | NR |  |  |

*Effect measure from which effectiveness estimate was derived

aIRR=adjusted incidence rate ratio; aHR=adjusted hazard ratio

^1^Poisson regression models adjusted for enrolment group (catch-up and seasonal), sex, and health district area

^2^Cox proportional hazards models adjusted for enrolment group (catch-up and seasonal), sex, and health district area

^3^Cox multivariate regression models were adjusted for any confounders with a standardized mean difference (SMD) between nirsevimab and control groups of >0.1

^4^adjusted for the week of the paediatric emergency department visit

**Table 5: Subgroup data: birth versus catch-up**

| Study | Outcome | Group | Follow-up | Events/ Sample size (incidence) | Effect measure* | Effectiveness |  |
| --- | --- | --- | --- | --- | --- | --- | --- |
|  |  |  |  |  | Estimate (95% CI) | Estimate (95% CI) |  |
| Ares-Gómez 2024 (cohort study) | RSV- LRTI hospitalisation | At birth nirsevimab | Median 81 days (IQR 68-87) | 15/3,188 (0.5%) | aIRR 0.36 (0.10, 2.27)^1^ | NR |  |
|  |  | At birth no nirsevimab |  | 2/152 (1.3%) |  |  |  |
|  |  | Catch-up nirsevimab |  | 15/6,220 (0.2%) | aIRR 0.15 (0.07, 0.31)^1^ | 85.35 (69.29, 92.96)^1^ |  |
|  |  | Catch-up no nirsevimab |  | 14/699 (2.0%) |  |  |  |
|  | RSV-LRTI with ICU admission | At birth nirsevimab | Median 81 days (IQR 68-87) | 7/3,188 (0.2%) | NR | NR |  |
|  |  | At birth no nirsevimab |  | 0/152 (NA) |  |  |  |
|  |  | Catch-up nirsevimab |  | 3/6,220 (0.2%) | NR | NR^2^ |  |
|  |  | Catch-up no nirsevimab |  | 0/699 (2.0%) |  |  |  |
| Ares-Gómez 2024 (cohort study) | | All-cause LRTI  hospitalisation | At birth nirsevimab | Median 81 days (IQR 68-87) | 85/3,186 (2.7%) | aIRR 0.41 (0.23, 0.85)^1^ | 58.7 (15.15 to 77.47)^1^ |
|  |  |  | At birth no nirsevimab |  | 10/154 (6.5%) |  |  |
|  |  |  | Catch-up nirsevimab |  | 65/6,201 (1.0%) | aIRR 0.27 (0.18, 0.42)^1^ | 72.95 (58.41 to 82.07)^1^ |
|  |  |  | Catch-up no nirsevimab |  | 33/715 (4.6%) |  |  |
|  |  | All-cause hospitalisation | At birth nirsevimab | Median 81 days (IQR 68-87) | 161/3,184 (5.1%) | aIRR 0.61 (0.36, 1.13)^1^ | NR |
| Lopez-Lacort 2024b (case-control study) | | RSV-LRTI primary care attendance | At birth cases | NA | 6/7 (NA) | NR | NR |
|  |  |  | At birth controls |  | 23/25 (NA) |  |  |
|  |  |  | Catch-up cases |  | 27/37 (NA) | NR | 80.2 (44.3 to 95.4) |
|  |  |  | Catch-up controls |  | 85/91 (NA) |  |  |

*Effect measure from which effectiveness estimate was derived

aHR=adjusted hazard ratio; aOR=adjusted odds ratio

^1^Calculated using Poisson regression models which were adjusted for sex, and health district area

^2^Effect measures and effectiveness could also not be reported due to 0 events for outcomes of ‘RSV-related LRTI with non-invasive mechanical ventilation’ and ‘RSV-related LRTI with invasive mechanical ventilation’. Effect measure and effectiveness not reported for ‘RSV-related LRTI hospitalisation with oxygen Support’

**Table 6: Subgroup data by age**

| Study | Outcome | Group | Follow-up | Events/ Sample size (incidence) | Effect measure* | Effectiveness |
| --- | --- | --- | --- | --- | --- | --- |
|  |  |  |  |  | Estimate (95% CI) | Estimate (95% CI) |
| Aguera 2024 (case-control study) | RSV-bronchiolitis related hospitalisation | Case: 0-3M | NA | NR | aOR NR | 78.2% (42.8–91.7)^1^ |
|  |  | Control: 0-3M | NA | NR |  |  |
|  |  | Case: 3-6M | NA | NR | aOR NR | 85.3% (22.5–97.2)^1^ |
|  |  | Control: 3-6M | NA | NR |  |  |
| Assad 2024 (case-control study) | RSV-bronchiolitis related hospitalisation | Case: 0-3M | NA | 53/332 (NA)^2^ | aOR NR | 82.4 (69.3 to 89.9)^3^ |
|  |  | Control: 0-3M | NA | 76/150 (NA)^2^ |  |  |
|  |  | Case: 3-6M | NA | 7/358 (NA)^2^ | aOR NR | 82.7 (52.8 to 93.7)^3^ |
|  |  | Control: 3-6M | NA | 21/195 (NA)^2^ |  |  |
| Carbajal 2024 (case-control study) | RSV-bronchiolitis related hospitalisation | Case: 0-3M | NA | 20/87 (NA)^2^ | NR | 78 (62 to 88)^4^ |
|  |  | Control: 0-3M | NA | NR |  |  |
|  |  | Case: 3-6M | NA | 2/66 (NA)^2^ | NR | 88 (71 to 97)^4^ |
|  |  | Control: 3-6M | NA | NR |  |  |
|  |  | Case: 6-12M | NA | 0/39 (NA)^2^ | NR | 89 (72 to 97)^4^ |
|  |  | Control >6M | NA | NR |  |  |
| Lassaued 2024 (case-control study) | RSV-bronchiolitis ambulatory care attendance | Case: 0-3M | NA | NR | NR | 65.5 (-0.8 to 94.0)^5^ |
|  |  | Control: 0-3M | NA | NR |  |  |
|  |  | Case: 3-6M | NA | NR | NR | 87.8 (66.9 to 95.5)^5^ |
|  |  | Control: 3-6M | NA | NR |  |  |
|  |  | Case: >6M | NA | NR | NR | 82.0(62.2 to 91.5)^5^ |
|  |  | Control: >6M | NA | NR |  |  |

*Effect measure from which effectiveness estimate was derived

aHR=adjusted hazard ratio; aOR=adjusted odds ratio

^1^Adjusted for age, weight, and the presence of at least one preexisting condition as fixed effects; the month of admission and hospital were treated as random effects.

^2^Events are the number of infants who had been given nirsevimab. The sample size is the number or infants in the case or control group. Incidence cannot be calculated for case-control studies.

^3^Multivariate regression model was adjusted for sex, gestational age at birth (as a continuous variable), birth weight, and risk factors for severe bronchiolitis

^4^adjusted for the week of the paediatric emergency department visit

^5^Multivariate regression model was adjusted for age, sex, birth term, birth weight, previous bronchiolitis, number of children per household, month of diagnosis, childcare settings, and region

**Table 7: Subgroup data: term versus pre-term**

| Study | Outcome | Group | Follow-up | Events/ Sample size (incidence) | Effect measure* | Effectiveness |
| --- | --- | --- | --- | --- | --- | --- |
|  |  |  |  |  | Estimate (95% CI) | Estimate (95% CI) |
| Aguera 2024 | RSV-bronchiolitis related hospitalisation: term | Case: term | NA | NR | NR | NR |
|  |  | Control: term | NA | NR |  |  |
|  |  | Case: pre-term | NA | NR | NR | 98.9 (33–100)^1^ |
|  |  | Control: pre-term | NA | NR |  |  |
| Lassaued 2024 (case-control study) | RSV-bronchiolitis ambulatory care attendance | Case: term | NA | NR | NR | 77.7 (62.5 to 86.8)^2^ |
|  |  | Control: term | NA | NR |  |  |
|  |  | Case: pre-term | NA | NR | NR | 56.6 (-1.2 to 92.5)^2^ |
|  |  | Control: pre-term | NA | NR |  |  |

*Effect measure from which effectiveness estimate was derived

^1^adjusted for age, weight, and the presence of at least one preexisting condition as fixed effects; the month of admission and hospital were treated as random effects

^2^Multivariate regression model was adjusted for age, sex, birth term, birth weight, previous bronchiolitis, number of children per household, month of diagnosis, childcare settings, and region

**Table 8: Subgroup data: 1^st^ versus 2^nd^ RSV season**

| Study | Outcome | Group | Follow-up | Events/ Sample size (incidence) | Effect measure* | Effectiveness (%) |
| --- | --- | --- | --- | --- | --- | --- |
|  |  |  |  |  | Estimate (95% CI) | Estimate (95% CI) |
| Lefferts 2024 (case control) | Medically attended^1^ RSV-ARIs: | Case: 1^st^ season (<8M) | NA | 8/39 (NA) | NR | 76 (42–90)^2^ |
|  |  | Control: 1^st^ season (<8M) | NA | 153/253 (NA) |  |  |
|  |  | Case: 2^nd^ season (>8M) | NA | 2/29 (NA) | NR | 88 (48-97)^2^ |
|  |  | Control: 2^nd^ season (>8M) | NA | 64/151 (NA) |  |  |

*Effect measure from which effectiveness estimate was derived

^1^hospitalisation, emergency department consultation or outpatient clinic visits

^2^Regression models were adjusted for age in months at medical visit, sex, calendar month, residence community type, and presence of one or more high-risk underlying condition
